# Efficacy of stem cell therapy for achilles tendon rupture: a systematic review and meta-analysis based on animal studies

**DOI:** 10.3389/fbioe.2026.1753018

**Published:** 2026-06-01

**Authors:** Bingbing Li, Xiaoyan Wang, Jian Ma, Dong Hu, Liang Liu, Jun Lu, Taichao Ren

**Affiliations:** 1 Department of Foot and Ankle Surgery, Honghui Hospital Affiliated to Xi’ an Jiaotong University, Xi’An, Shaanxi, China; 2 Hospital of Northwestern Polytechnical University, Xi’An, Shaanxi, China

**Keywords:** achilles tendon rupture, animal studies, biomaterials, meta-analysis, stem cells

## Abstract

**Background:**

Although stem cell–based therapies show promise for promoting Achilles tendon regeneration, the current preclinical evidence is highly heterogeneous, and their overall efficacy and key modifying factors remain unclear.

**Methods:**

We systematically searched PubMed, Web of Science, Embase, and Scopus for studies based on rat models of Achilles tendon rupture. Two investigators independently performed study selection, data extraction, and risk-of-bias assessment. Meta-analyses were conducted using R software.

**Results:**

Twenty-seven studies were included. Pooled analyses showed that stem cell transplantation significantly improved ultimate load and histological scores of the Achilles tendon, with effects increasing over time. However, the impact of stem cell therapy on tendon stiffness was limited. For type I collagen, stem cell transplantation alone did not significantly enhance its expression, whereas combining stem cells with biomaterial scaffolds led to a marked increase. Subgroup analyses revealed a complex relationship between stem cell dose and treatment efficacy: for ultimate load, a moderate dose was effective at 2 weeks, while a high dose was more effective at 4 weeks; for histological scores, both low and moderate doses were effective at 2 weeks, whereas only moderate-to-high doses were effective at 4 weeks, indicating a nonlinear and time-dependent dose–response pattern. Overall, composite strategies using scaffold-loaded stem cells were generally more effective than scaffold treatment alone. Assessment of publication bias suggested potential bias in the primary outcomes, but trim-and-fill correction did not materially alter the conclusions. Risk-of-bias evaluation showed generally inadequate reporting in key methodological domains, including random sequence generation, allocation concealment, and blinding, indicating a potential risk of bias.

**Conclusion:**

Stem cell transplantation can effectively improve the biomechanical strength and histological architecture of the Achilles tendon after rupture, but its effects on stiffness restoration and type I collagen expression appear limited. Based on early healing data from rat models, scaffold-loaded stem cell therapy shows potential for enhancing Achilles tendon repair. However, the current preclinical literature is characterized by methodological shortcomings and marked heterogeneity in critical factors such as stem cell sources and scaffold materials. As a result, the findings primarily reflect overall trends rather than definitive effects, and clinical translation will require further standardized studies and validation in large animal models.

## 1 Background

The Achilles tendon, as the largest tendon in the human body, plays a crucial role in activities such as walking and running; however, it is also prone to injury. The incidence of Achilles tendon rupture is approximately 11–37 cases per 100,000 individuals, with a notable upward trend in recent years, particularly among men aged 35 to 55 who engage in intermittent high-intensity sports (Millar et al., 2021a; Russo et al., 2022; Park et al., 2020; Lemme et al., 2018). Achilles tendon ruptures not only result in significant pain and functional impairment but also often heal poorly due to inadequate blood supply and limited self-repair capacity, leading to complications such as re-rupture and infection (Millar et al., 2021a; Van Schaik and Lee, 2021). Although current surgical and conservative treatments can restore the anatomical continuity of the tendon, the healing process frequently results in the formation of excessive scar tissue, which significantly diminishes the biomechanical properties of the tendon and makes it difficult to restore its original structure and function (Baltes et al., 2017; Migliorini et al., 2020). Furthermore, the Achilles tendon itself has a poor blood supply and weak self-repair capabilities, a problem that is particularly exacerbated by persistent local inflammatory responses, abnormal proliferation and differentiation of fibroblasts, and disorganized collagen fiber arrangement following rupture, all of which contribute to delayed healing and functional impairment (Heikkinen et al., 2017). Therefore, it is paramount to explore novel therapeutic strategies that can promote tendon regeneration and improve tendon biomechanical properties (Millar et al., 2021a; Millar et al., 2021b).

In recent years, stem cell therapy has emerged as a promising avenue for tendon repair due to its multipotent differentiation potential and robust paracrine effects. Mesenchymal stem cells derived from bone marrow, adipose tissue, and tendon can aid in tendon regeneration through various mechanisms, including differentiation into tenocyte-like cells, secretion of active factors to modulate the microenvironment, and promotion of organized collagen alignment. The stem cell–scaffold composite system, which combines stem cells with biomaterial scaffolds, can better mimic the natural extracellular matrix by enhancing cell retention and promoting directed differentiation efficiency, thus showing great promise (Ding et al., 2022). Notably, stem cell–derived exosomes have emerged as a novel acellular therapeutic strategy and have attracted increasing attention in tendon regeneration research in recent years, representing an important extension of stem cell–based approaches (Zhang et al., 2023; Kasula et al., 2024). However, despite the enthusiasm for preclinical research, the evidence base in this field exhibits significant heterogeneity and inconsistency. This heterogeneity is prevalent across various dimensions, including the selection of cell sources, delivery strategies (e.g., use alone or in combination with various biomaterial scaffolds), and the timing and standards for efficacy assessment (Bowers et al., 2022; Ho et al., 2014; Jiang et al., 2023; Wang et al., 2024; Yea et al., 2023). These methodological discrepancies not only hinder direct comparisons of study conclusions but also pose substantial challenges in identifying the most translationally promising treatment options. Therefore, this study aims to conduct a rigorously designed systematic review and meta-analysis to quantitatively evaluate the overall effects of stem cell interventions on key outcome measures—biomechanical properties, histological characteristics, and molecular markers—in rat models of Achilles tendon rupture. Furthermore, through detailed subgroup analyses, we seek to investigate how critical variables such as the mode of stem cell delivery, therapeutic time window, and transplantation dosage influence treatment efficacy. The ultimate goal is to provide evidence-based guidance for the standardized design of future preclinical studies and to inform the development of protocols for large-animal experiments and early-phase clinical trials.

## 2 Methods

### 2.1 Study design and registration

This study has been prospectively registered with the International Prospective Register of Systematic Reviews (PROSPERO) under registration number CRD420251087206. The design, implementation, and reporting of the study strictly adhere to the Preferred Reporting Items for Systematic Reviews and Meta-Analyses (PRISMA) guidelines, as well as the guidelines for systematic reviews of animal studies, to ensure methodological rigor and result reliability.

### 2.2 Inclusion and exclusion criteria

#### 2.2.1 Study type

Only published randomized controlled trials (RCTs) were included, regardless of whether allocation concealment or blinding was employed. Non-randomized controlled studies (e.g., self-comparison studies) were excluded.

#### 2.2.2 Study subjects

Only studies using rats as experimental animals were included, without restrictions on rat strains (e.g., SD rats, Wistar rats), sex, or age, provided that baseline characteristics (e.g., weight, age) were clearly reported. The animal model must be an acute Achilles tendon rupture model (established through surgical transection of the tendon, such as a transverse cut in the mid-tendon), excluding models of tendinitis, tendon degeneration, and other tendon pathologies.

#### 2.2.3 Interventions

The experimental group must include stem cell transplantation, specifically: ① standalone stem cell transplantation (e.g., BMSC, ADMSC, TSC, etc., with no restrictions on stem cell source or extraction method); ② stem cell transplantation combined with scaffold materials, with no restrictions on scaffold types, such as collagen scaffolds or poly (lactic-co-glycolic acid) scaffolds. The control group must provide a clear comparison with the experimental group, including: ① blank control (only tendon rupture, without other interventions); ② vehicle control (e.g., injection of saline or phosphate-buffered saline as stem cell carriers, or implantation of scaffold materials alone); ③ comparisons of different stem cell types/doses (e.g., BMSC vs. ADMSC, low-dose stem cells vs. high-dose stem cells), ensuring that the control group does not contain stem cell components (except for scaffold-only controls).

#### 2.2.4 Outcome measures

Studies were required to report extractable data for at least one of the following outcomes: (1) Biomechanical outcomes: The primary focus of this study was on parameters reflecting the overall structural and mechanical performance of the tendon, including ultimate load (the maximum tensile force the Achilles tendon can withstand at the point of failure, usually expressed in N) and stiffness (the ratio of force to deformation during loading, expressed in N/mm). It should be noted that tendons exhibit a characteristic nonlinear stress–strain behavior, and stiffness values depend on the specific segment of the load–displacement curve selected for analysis. The methods used to measure stiffness varied across the included studies: most defined stiffness as the slope of the linear portion of the load–displacement curve (i.e., linear stiffness), whereas some used secant stiffness within a predefined displacement range or the maximum slope of the entire curve. To maintain data consistency, we extracted stiffness values as defined in the original reports and pooled them for analysis, while fully accounting for this methodological heterogeneity in the interpretation of the results. Material property parameters normalized for tendon geometry, such as ultimate tensile strength (UTS, MPa) and elastic modulus (MPa or N/mm^2^), were reported infrequently and thus were not included in the meta-analysis due to insufficient data for a robust pooled estimate. (2) Histological outcomes: Histological scores (assessed using established scoring systems, such as the Bonar score or modified tendon healing scores) were used to evaluate collagen fiber organization, cell density, inflammatory response, and other aspects of Achilles tendon healing. (3) It should be noted that, based on the characteristics of the original data reported and in order to preserve statistical power for subgroup analyses (by time point, intervention type, and dose), we combined gene expression (mRNA level) and protein expression data in the meta-analysis and treated them as a composite indicator reflecting the overall expression level of type I collagen.

#### 2.2.5 Exclusion criteria

① Non-English publications; ② Studies for which full texts could not be obtained, or where original data (e.g., mean, standard deviation, sample size for outcome measures) could not be acquired even after contacting the authors; ③ Autologous control studies (e.g., using bilateral Achilles tendons of the same rat as experimental and control groups, which pose risks of stem cell migration, exercise interference, and pain stress); ④ Non-original research such as reviews, meta-analyses, case reports, commentaries, conference abstracts, and preprints; ⑤ Studies that do not clearly report the methods for establishing the Achilles tendon rupture model, the type of stem cells used, or the timing of outcome measure assessments.

### 2.3 Literature search strategy

To comprehensively identify all relevant literature, we systematically searched databases including PubMed, Web of Science, Embase, and Scopus, with the search timeframe extending from the inception of each database to 9 January 2026. The search strategy was constructed using a combination of subject headings and free-text terms, tailored and optimized according to the indexing rules of each database, including terms such as “Achilles tendon,” “stem cells,” and their corresponding synonyms and related terms. Additionally, to minimize the risk of missing relevant studies, we implemented supplementary manual searches by systematically reviewing the reference lists of published related reviews. The specific search queries are detailed in Supplementary Table S1.

### 2.4 Literature screening and data extraction

The literature screening and data extraction processes were independently conducted in parallel by two researchers trained in systematic review methodology, with any discrepancies resolved through consensus or consultation with a third senior researcher. The screening process employed a tiered approach, initially excluding clearly irrelevant studies based on titles and abstracts, followed by a full-text review of potentially eligible studies to finalize inclusion. Data extraction utilized a pre-designed and piloted standardized electronic spreadsheet, capturing comprehensive information on study characteristics, baseline animal features, model construction details, specific parameters of interventions and controls, and endpoint data for all predefined outcome measures (means and standard deviations). In cases of incomplete data reporting, we contacted the corresponding authors of the original studies via email to obtain the original data.

### 2.5 Risk of bias assessment

To evaluate the internal validity of the included studies, we employed the SYRCLE Risk of Bias tool specifically designed for animal studies. This tool assesses ten key domains: sequence generation, allocation concealment, baseline characteristics, random housing, blinding of personnel, random outcome assessment, blinding of outcome assessment, incomplete outcome data, selective reporting, and other biases. Two researchers conducted independent assessments, with each item rated as “yes” (low risk of bias), “no” (high risk of bias), or “unclear” (insufficient information). Final consensus was reached through discussion, systematically revealing the methodological quality of the included studies.

### 2.6 Statistical analysis

All statistical analyses were conducted using R statistical software (version 4.4.2), utilizing packages such as meta, metafor, dplyr, ggplot2, and forestplot for data processing and visualization. In this study, all outcome measures were treated as continuous variables, and the Standardized Mean Difference (SMD) along with its 95% Confidence Interval (95% CI) was employed as the effect size to account for differences in measurement units across studies. Heterogeneity among studies was quantitatively assessed using Cochran’s Q test (with a significance level set at α = 0.10) and the I^2^ statistic: ① If I^2^ ≤ 50% and Q test P ≥ 0.10, heterogeneity was considered low, and a fixed-effect model was used for the meta-analysis; ② If I^2^ > 50% or Q test P < 0.10, indicating substantial heterogeneity, a random-effects model (using restricted maximum likelihood estimation to assess between-study variance τ^2^) was employed for the combined analysis, followed by subgroup analyses to explore the sources of heterogeneity.

To investigate the impact of intervention strategies on efficacy, the following predefined subgroup analyses were conducted: by type of stem cell intervention: standalone stem cell transplantation group vs. stem cell + scaffold transplantation group or stem cell transplantation group vs. negative control group; by type of stem cell: BMSC group vs. ADMSC group vs. TSC group (if sufficient studies were included); by follow-up time points: 2-week group vs. 4-week group vs. 8-week group; and by stem cell transplantation dosage: low-dose group (e.g., < 1 × 10^6^ cells) vs. medium-dose group (1 × 10^6^ cells) vs. high-dose group (e.g., > 1 × 10^6^ cells, with specific dosage group boundaries adjusted based on the actual data from included studies).

For the outcome measure with the highest number of included studies, the following methods were used to assess publication bias: ① Funnel Plot: A funnel plot was created with effect size (SMD) on the x-axis and standard error (SE) on the y-axis, visually assessing symmetry (asymmetry in the funnel plot typically indicates the presence of publication bias); ② Egger’s linear regression test and Begg’s rank correlation test: A P-value < 0.05 was considered statistically significant for determining the presence of publication bias. If publication bias was detected, the trim-and-fill method was employed for correction, to evaluate the impact of bias on the combined effect size and verifying the robustness of the conclusions. Sensitivity analysis was performed by switching between the fixed-effect and random-effects models to compare the differences in results. If the sensitivity analysis results were consistent with the original findings, it indicated robustness; if there were significant discrepancies, further analysis of the reasons would be warranted.

## 3 Results

### 3.1 Literature search results

**FIGURE 1:**
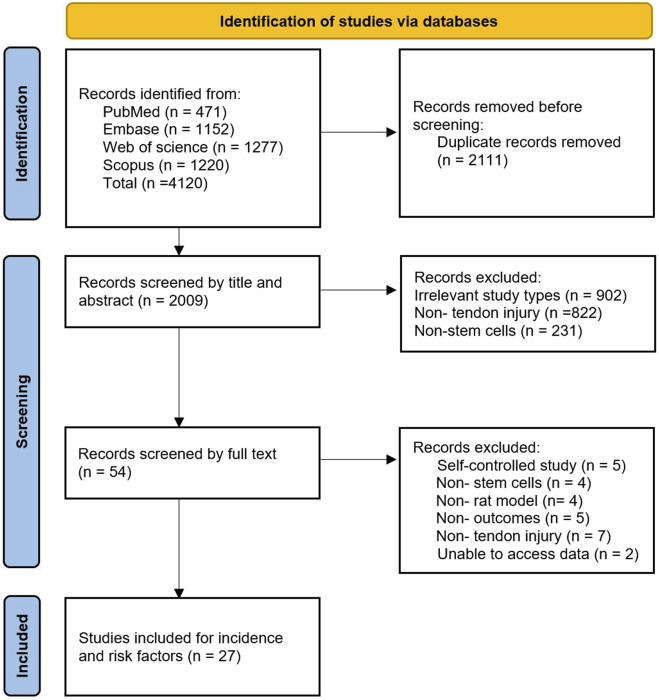
Flowchart of the literature screening process.

A total of 4,120 relevant articles were identified in the initial search. After removing 2,111 duplicate records, 2,009 articles were screened based on titles and abstracts, resulting in the exclusion of 1,995 articles. Following a full-text review of the remaining 54 articles, 27 studies met all inclusion criteria and were included in this systematic review and meta-analysis (Figure 1). All included studies were randomized controlled animal experiments published between 2010 and 2025.

### 3.2 Basic characteristics of included studies

The 27 studies involved various rat strains, including Sprague-Dawley (SD) rats (16 studies), Wistar rats (7 studies), Lewis rats (2 studies), athymic nude rats (1 study), and Fischer rats (1 study). The majority of the animals were male (17 studies), while 7 studies included females, 2 did not report sex, and 1 did not report sex information. The weight of the rats ranged from 190 to 410 g (5 studies did not report weight), and the age ranged from 6 to 18 weeks (10 studies did not report age). All studies utilized a surgical transection method to establish an acute Achilles tendon rupture model. The types of stem cells used included adipose-derived mesenchymal stem cells (ADMSC, 9 studies), bone marrow-derived mesenchymal stem cells (BMSC, 12 studies), human umbilical cord-derived mesenchymal stem cells (hUCMSC, 1 study), induced pluripotent stem cell-derived mesenchymal stem cells (iMSC, 1 study), and tendon-derived stem cells (TDSC, 5 studies), with one study using both BMSC and TDSC. In terms of intervention strategies, 12 studies employed biomaterial scaffolds to assist stem cell transplantation, while 15 studies involved local injection of stem cells. Post-operative follow-up durations ranged from 2 to 16 weeks, and the sample sizes varied from 6 to 90 animals. Detailed study characteristics are presented in Supplementary Table S2.

### 3.3 Risk of bias assessment results

**FIGURE 2:**
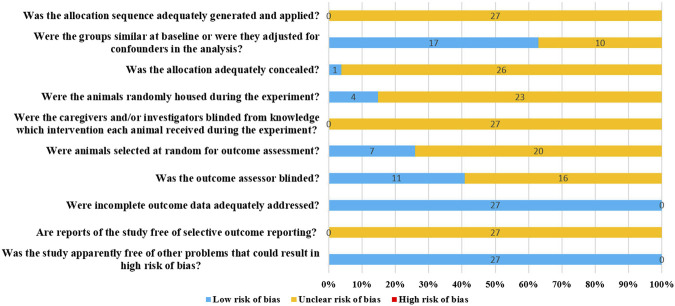
Results of the risk of bias assessment.

All studies exhibited low risk regarding “other sources of bias” and “incomplete outcome data.” Most studies (17 studies) also demonstrated low risk concerning “baseline characteristics.” However, the majority of studies were deemed to have “unclear risk of bias” for key methodological items such as “random sequence generation,” “allocation concealment,” “random housing,” “blinding of personnel,” “blinding of outcome assessment,” and “random outcome assessment” due to insufficient reporting. The “selective reporting” item also indicated potential risk across all studies due to unclear information. Overall, the reporting quality of randomization and blinding implementation in the included studies requires improvement, indicating a potential risk of bias. Detailed results of the risk of bias assessment are shown in Figure 2.

### 3.4 Meta-analysis results

#### 3.4.1 Ultimate load

**FIGURE 3:**
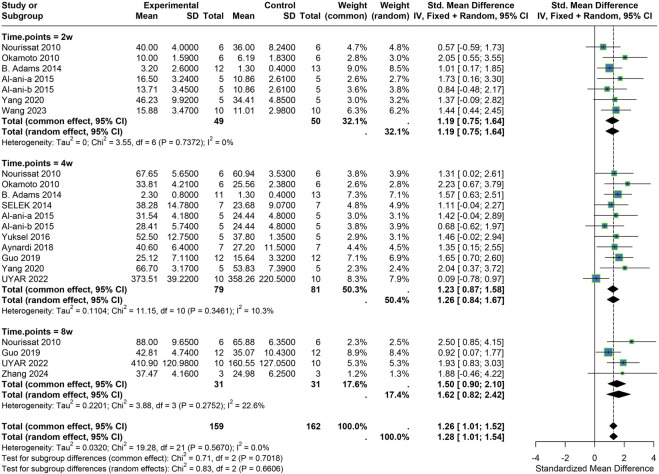
Ultimate load comparison between stem cells and control groups.

A total of 21 studies reported on ultimate load. Compared to the negative control group, stem cell transplantation significantly increased the ultimate load of the Achilles tendon (fixed-effect model, Figure 3), with efficacy increasing over time. When compared to scaffold implantation alone, scaffolds loaded with stem cells demonstrated greater improvement (fixed-effect model, Supplementary Figure S1), with this advantage also increasing over time.

Subgroup analysis by dosage indicated that at 2 weeks post-surgery, moderate-dose stem cell transplantation significantly improved ultimate load, while low and high-dose groups did not show significant advantages (Supplementary Figure S2). At 4 weeks, all dosage groups significantly outperformed the control group, with effect strength following the trend of high-dose > low-dose > moderate-dose (Supplementary Figure S3). In the scaffold-assisted group, at 2 weeks post-surgery, both low and moderate doses of stem cells outperformed the scaffold alone, with the low dose showing better effects (Supplementary Figure S4). At 4 weeks, all dosage groups loaded with stem cells significantly surpassed the scaffold-only group, with effect strength following the trend of low-dose > high-dose > moderate-dose (Supplementary Figure S5).

#### 3.4.2 Stiffness

**FIGURE 4:**
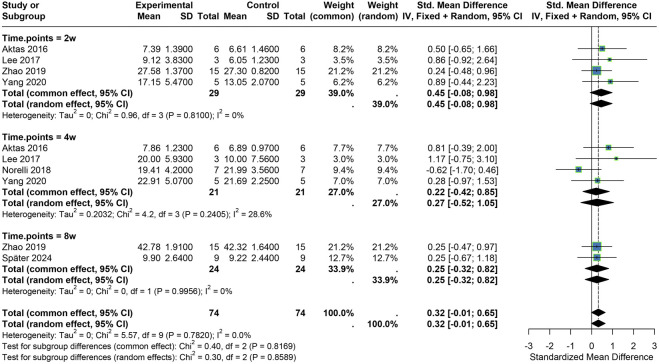
Stiffness comparison between Scaffold + Stem Cells and Scaffold groups.

Eight studies reported on stiffness metrics. Random-effects model meta-analysis revealed that stem cell transplantation did not significantly improve Achilles tendon stiffness compared to the negative control group at 2, 4, or 8 weeks (Supplementary Figure S6). Similarly, scaffolds loaded with stem cells did not show statistically significant advantages over scaffold-only transplantation (Figure 4). In the dosage subgroup analysis, only high-dose stem cell transplantation at 4 weeks showed a statistically significant advantage (Supplementary Figure S7), while no significant differences were observed at other time points or dosage groups (Supplementary Figures S7-S9).

#### 3.4.3 Histological scores

**FIGURE 5:**
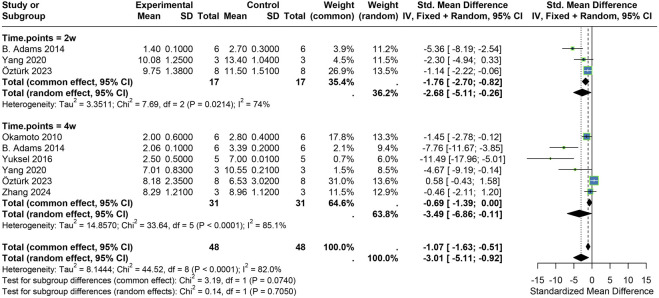
Histological scores comparison between stem cells and control groups.

Twelve studies utilized histological scoring to assess tendon structural repair. Random-effects model analysis indicated that stem cell transplantation significantly improved histological scores (Figure 5), with efficacy increasing over time. The histological improvement in the scaffold-loaded stem cell group was also significantly better than that in the scaffold-only group (Supplementary Figure S10), exhibiting a time-dependent enhancement.

Dosage analysis showed that at 2 weeks post-surgery, both low and moderate doses of stem cell transplantation effectively improved histological scores, with moderate doses showing superior effects (Supplementary Figure S11). By 4 weeks, both moderate and high-dose groups demonstrated significant advantages, with moderate doses being the most effective, while the low-dose group did not reach statistical significance (Supplementary Figure S12). In the scaffold-assisted group, at both 2 and 4 weeks post-surgery, the low-dose stem cell group significantly outperformed the scaffold-only group, while the moderate-dose stem cell group did not show significant advantages (Supplementary Figures S13, S14).

#### 3.4.4 Type I collagen

**FIGURE 6:**
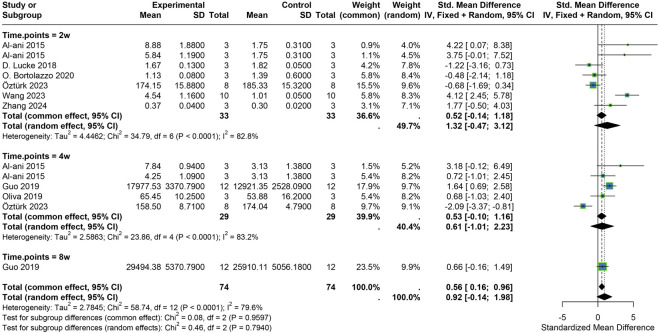
Type I collagen expression comparison between stem cells and control groups.

A total of 12 studies reported data on type I collagen expression. Given the characteristics of the original data and to preserve statistical power for subsequent subgroup analyses (by time point, intervention type, and dose), we pooled gene expression (mRNA level) and protein expression data and treated them as a composite indicator of overall type I collagen expression. Random-effects meta-analysis showed that stem cell transplantation did not significantly increase type I collagen expression at any time point (Figure 6). In contrast, type I collagen expression in the scaffold-loaded stem cell group was significantly higher than in the scaffold-only group (fixed-effects model, Supplementary Figure S15), and this effect became more pronounced over time. The scaffolds included in this analysis were poly (lactide-co-glycolide) (PLG) scaffolds, poly (lactide-co-glycolic acid) (PLGA) scaffolds, collagen scaffolds, and collagen/alginate hydrogel scaffolds. Because each scaffold type was represented by only a single study, subgroup analyses stratified by scaffold material could not be performed.

Dosage analysis revealed that compared to the negative control, only moderate-dose stem cells at 2 weeks and high-dose stem cells at 4 weeks significantly enhanced type I collagen expression (Supplementary Figures S16, S17). In the scaffold-assisted group, at 2 weeks post-surgery, neither low nor moderate doses of stem cells showed significant advantages (Supplementary Figure S18). The results of the subgroup analyses are presented in Table 1.

**TABLE 1:** Summary of outcome indicators.

Subgroup	Time points	Dose	Outcome measure	Effect size (SMD, 95% CI)	Model (heterogeneity)
Stem cells vs. Negative contro	2 Weeks	High dose	Ultimate load	1.23 [-0.21, 2.67]	Random effects (I^2^ = 57.1%)
		Moderate dose	Ultimate load	1.19 [0.65, 1.74]	Fixed effects (I^2^ = 0.0%)
		Low dose	Ultimate load	1.37 [-0.09, 2.82]	Single study
		Moderate dose	Histological scores	−5.36 [-8.19, −2.54]	Single study
		低/Moderate dose	Histological scores	−2.68 [-5.11, −0.26]	Random effects (I^2^ = 74.0%)
		Moderate dose	Type I collagen	2.31 [1.53, 3.09]	Fixed effects (I^2^ = 0.0%)
	4 Weeks	High dose	Ultimate load	1.65 [0.70, 2.60]	Fixed effects (I^2^ = 0.0%)
		Moderate dose	Ultimate load	1.34 [0.87, 1.82]	Fixed effects (I^2^ = 0.0%)
		Low dose	Ultimate load	1.35 [0.15, 2.55]	Fixed effects (I^2^ = 0.0%)
		Moderate dose	Histological scores	−8.76 [-12.10, −5.41]	Fixed effects (I^2^ = 0.0%)
		High dose	Histological scores	−1.06 [-2.10, −0.02]	Fixed effects (I^2^ = 0.0%)
		Low dose	Histological scores	0.58 [-0.43, 1.58]	Fixed effects (I^2^ = 79.6%)
		High dose	Type I collagen	1.42 [0.59, 2.24]	Fixed effects (I^2^ = 0.0%)
		High dose	Stiffness	0.90 [0.05, 1.74]	Random effects (I^2^ = 74.9%)
Scaffold + Stem cells vs. Scaffold alone	2 Weeks	Low dose	Ultimate load	1.72 [0.15, 3.28]	Single study
		Moderate dose	Ultimate load	1.06 [0.03, 2.09]	Fixed effects (I^2^ = 0.0%)
		Low dose	Histological scores	−2.72 [-4.79, −0.65]	Random effects (I^2^ = 13.0%)
	4 Weeks	Low dose	Ultimate load	1.80 [0.21, 3.39]	Single study
		Moderate dose	Ultimate load	1.47 [0.05, 2.89]	Random effects (I^2^ = 52.4%)
		High dose	Ultimate load	1.49 [0.57, 2.40]	Fixed effects (I^2^ = 0.0%)
		Low dose	Histological scores	−3.80 [-7.59, 0.00]	Random effects (I^2^ = 11.6%)

#### 3.4.5 Publication bias detection

**FIGURE 7:**
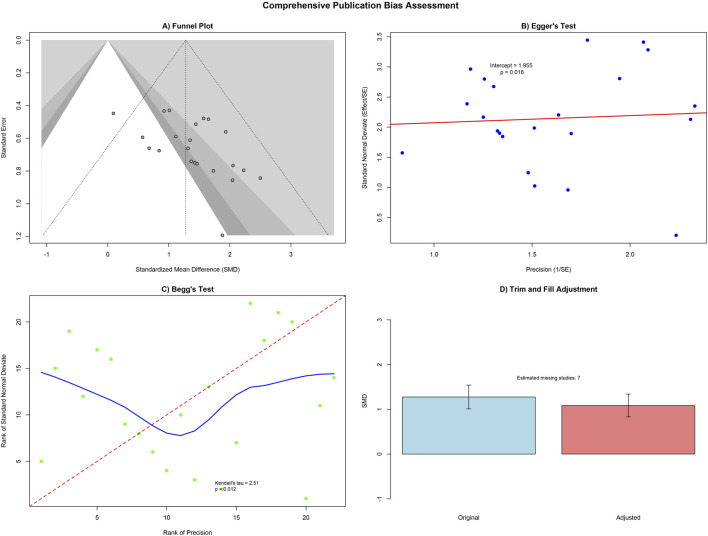
Publication bias assessment for ultimate load comparison between stem cells and control groups. **(A)** Funnel plot **(B)** Egger’s test **(C)** Begg’s test **(D)** Trim and fill adjustment.

Publication bias was assessed for the outcome measure with the highest number of studies, ultimate load (n = 21). The funnel plot for the stem cell intervention group vs. the negative control group displayed an asymmetric distribution (Figure 7A), and both Egger’ s (Figure 7B) and Begg’s (Figure 7C) tests indicated potential publication bias. After correction using the trim-and-fill method, an estimated 7 studies were found to be missing; however, the corrected combined effect size remained statistically significant (SMD = 1.086, 95% CI: 0.832–1.340), indicating the robustness of the results (Figure 7D). The funnel plot for the scaffold-loaded stem cell vs. scaffold-only group was nearly symmetric (Supplementary Figure S19A), and neither Egger’s (P = 0.215) nor Begg’s (P = 0.184) tests revealed significant publication bias, with the trim-and-fill analysis also not identifying any missing studies (Supplementary Figure S19D).

## 4 Discussion

This study is the first to systematically evaluate the comprehensive effects of stem cell therapy on biomechanical performance, histological structure, and molecular markers in a rat model of Achilles tendon rupture through a systematic review and meta-analysis. The results indicate that stem cell-based interventions can improve tensile strength and histological structure; however, their impact on stiffness and type I collagen enhancement is limited, suggesting that while structural repair benefits are evident, there remains a gap between structural restoration and functional recovery.

From a biomechanical perspective, stem cell transplantation significantly increased the ultimate load of the Achilles tendon, with efficacy progressively enhancing over the repair period. This finding aligns with the multiple biological mechanisms that stem cells exert in tendon repair. On one hand, stem cells possess the potential to differentiate into tenocyte-like cells; for instance, BMSC and TDSC can upregulate tendon-specific markers such as scleraxis and tenomodulin, promoting the synthesis and deposition of type I collagen, thereby enhancing the structural integrity of the tendon (Costa-Almeida et al., 2019; Guerquin et al., 2013; Kim et al., 2021; Yu et al., 2020). On the other hand, their paracrine functions may play a more central role. Stem cells secrete various growth factors, such as TGF-β, IGF-1, bFGF, and VEGF, which modulate local inflammatory responses, promote angiogenesis, and recruit endogenous repair cells, collectively guiding the repair process toward physiological “tendinous healing” rather than scar tissue formation (Costa-Almeida et al., 2019; Torres-Torrillas et al., 2019; Kao and Papoutsakis, 2019; Manning et al., 2015). The significant improvement in histological scores, such as more organized collagen fiber alignment and cell morphology and density closer to normal tendon structure, further corroborates the structural remodeling of the tendon extracellular matrix.

However, stem cell therapy did not significantly improve tendon stiffness, which may indicate that the restoration of mechanical properties remains incomplete. Stiffness, as a key indicator of a tissue’s resistance to deformation, is highly dependent on the alignment, maturity, and inter-fiber cross-linking of collagen fibers (Ishizaki et al., 2024). Moreover, He et al. (He et al., 2024) particularly emphasized that mechanical stimulation plays a decisive role in the differentiation of TDSC into functional tendon cells, and the lack of timely mechanical loading can lead to abnormal differentiation and incomplete matrix remodeling. In the early stages of healing, the repaired tissue primarily consists of thin and disorganized type III collagen, which has significantly lower mechanical properties compared to mature type I collagen fiber bundles (Schneider et al., 2018; Zhang et al., 2021; Li et al., 2023). Although stem cell therapy can promote collagen synthesis, the observation window in the current studies may not be sufficient to fully reconstruct the highly organized collagen ultrastructure and elastin network, leading to delayed stiffness recovery (Zhang et al., 2021; Li et al., 2023). This phenomenon suggests that a cell-based therapy alone still has limitations in reproducing the complex mechanical characteristics of normal tendons. Additionally, it is noteworthy that standalone stem cell transplantation did not significantly enhance type I collagen expression, while the combination with biomaterial scaffolds significantly promoted its expression, highlighting the critical role of scaffolds in constructing a biomimetic microenvironment. Biomaterial scaffolds not only provide three-dimensional support for cell attachment and growth, improving cell retention and viability in the injury area, but also actively guide cell differentiation and the synthesis and assembly of functional extracellular matrix through their biochemical composition (e.g., collagen, fibronectin) and topographical structure (Wang et al., 2025; Guo et al., 2021; Yin et al., 2020). For instance, collagen-based or decellularized matrix scaffolds can mimic the composition and microstructure of natural tendons, providing a template for collagen deposition and alignment (Deepthi et al., 2016; No et al., 2020). In contrast, injected stem cells may have low survival rates and limited tenogenic differentiation efficiency due to an unstable local mechanical environment, insufficient blood supply, and a pro-inflammatory microenvironment, thereby weakening their direct contribution to collagen synthesis (No et al., 2020).

It is important to emphasize that the increase in ultimate load observed in this study does not equate to full functional recovery of the tendon. Both ultimate load and stiffness are structural properties, describing the mechanical behavior of the entire tendon–bone complex as a structural unit. Their values depend not only on the intrinsic material properties of the tissue, but also on its geometric dimensions (e.g., cross-sectional area). As a result, an increase in ultimate load may result from an enlargement of the cross-sectional area of the repair tissue or an overall increase in collagen content, without indicating whether collagen fibers are highly aligned along the physiological loading direction. Similarly, an increase in stiffness may simply reflect an enlarged cross-sectional area, rather than genuine improvements in collagen fiber orientation and crosslink maturation (Logerstedt et al., 2022; Cop et al., 2021). By contrast, geometry-normalized material properties, such as ultimate tensile strength (UTS) and elastic modulus, more directly reflect the intrinsic quality of the tissue itself. In this study, although stem cell therapy significantly improved ultimate load, stiffness did not show a significant increase at most time points. This pattern may indicate that the repair tissue bears load primarily by increasing its volume, while its intrinsic material characteristics (e., collagen fiber alignment and crosslinking) still fall short of those of normal tendon. Unfortunately, because material property outcomes were sparsely reported in the included studies, we were unable to test this hypothesis directly. Therefore, our findings support viewing stem cell therapy more as a strategy that promotes structural repair and enhances load-bearing capacity, rather than as a solution that has already achieved complete functional regeneration. In addition, most studies had follow-up periods concentrated within 2–4 weeks postoperatively, corresponding to the early phase of healing. Structural and tensile improvements observed within this time window are unlikely to reflect the recovery of functional parameters at maturation, such as stiffness, viscoelastic behavior, and fatigue resistance, and should not be directly extrapolated to clinical scenarios in humans.

At the molecular level, the outcomes suitable for pooling in this study were mainly related to type I collagen. During data extraction, we also focused on markers of tenogenic differentiation, inflammatory/immune mediators, and growth factor–related signaling molecules. However, the specific indicators reported varied widely across studies, as did the level and format of measurement (transcriptional, protein, or semi-quantitative tissue staining), and some studies lacked complete statistical data (means, standard deviations, and sample sizes) required for quantitative synthesis. As a result, we were unable to perform meta-analyses for these targets. For type I collagen, the included studies used RT-PCR, Western blotting, and immunohistochemistry/immunofluorescence, capturing both gene and protein expression. Ideally, a meta-analysis should strictly separate these two levels and pool them individually to avoid conflating distinct biological layers of information.

In this study, however, further stratification by gene versus protein expression, combined with the predefined time points (2, 4, and 8 weeks), intervention types (stem cells vs. controls, scaffold-loaded stem cells vs. scaffold alone), and dose groups, would have resulted in too few studies per subgroup (in cases only 1–2), precluding meaningful pooling and robust statistical inference. Therefore, based on the available evidence and considering that the original studies generally treated type I collagen expression as an indicator of successful repair, we combined gene and protein expression data in the present analysis and used them as a composite measure of overall type I collagen expression. We fully recognize the limitations of this approach: gene and protein expression do not always change in parallel, and a pooled analysis may obscure differences between them. Consequently, the pooled effect sizes are best understood as a statistical summary of overall differences in type I collagen expression, rather than direct evidence of collagen structural maturation, and they are insufficient to infer translational efficiency or protein function. It must be stressed that changes in gene expression do not equate to changes in protein expression, and neither directly reflects the maturity of collagen fibril structure. Thus, alterations in expression—whether at the gene or protein level—cannot, on their own, directly indicate improvements in collagen fiber reorientation, crosslink maturation, or fibrillar ultrastructure, nor do they provide a sufficient basis for definitive conclusions regarding tenogenic differentiation, growth factor signaling, or immunomodulatory mechanisms. Future studies should adopt standardized and reproducible reporting formats that distinguish gene from protein expression, and should incorporate structural/ultrastructural assessments together with viscoelastic testing and mechanical evaluation within physiological loading ranges, in order to strengthen mechanistic interpretation and the assessment of functional recovery.

Interpretation of type I collagen expression also needs to take into account the temporal dynamics of tendon healing. Under physiological conditions, tendon repair involves a characteristic time-dependent collagen transition. In the early phase after injury, rapid deposition of type III collagen predominates, forming the initial fibrous bridge; as healing progresses, type III collagen is gradually replaced by type I collagen, which becomes the main collagen component of mature tendon (Ren et al., 2025; Rieu et al., 2017). In our review, most studies assessing type I collagen had follow-up periods concentrated at 2–4 weeks postoperatively (accounting for 55.56% of the studies included in the type I collagen analysis), corresponding exactly to the critical transitional phase of collagen remodeling. Therefore, the lack of a significant enhancement of type I collagen expression after stem cell transplantation observed in this study may be partly attributable to the relatively early evaluation time points. At this stage, the repair tissue is still dominated by type III collagen, and the orderly deposition and crosslinking of type I collagen have not yet fully commenced. This timing bias leads to an underestimation of the true effect of stem cells on promoting collagen maturation. Future studies should extend the follow-up period to at least 8 weeks to more accurately evaluate the impact of stem cell therapy on type I collagen deposition and maturation.

Further subgroup analyses highlighted the influence of stem cell type, transplantation dose, and delivery strategy on treatment efficacy. With respect to cell source, BMSCs, ADMSCs, and TDSCs were all represented in the included studies. TDSCs, owing to their inherent tenogenic differentiation propensity, have been reported to exhibit superior repair effects compared to other cell types in some studies (Al-Ani et al., 2015; Guo et al., 2016; Youngstrom et al., 2016). However, the number of head-to-head comparative studies is currently insufficient to identify an optimal cell type, and more direct comparison trials are needed. Regarding dose–response relationships, the data revealed a nonlinear pattern. For ultimate load, moderate doses (e.g., 1 × 10^6^ cells) produced significant improvements at early time points (2 weeks), whereas high doses showed more pronounced effects at later stages (4 weeks), suggesting that excessively high doses may cause local cellular crowding within the microenvironment and intensified competition for nutrients, thereby impairing cell survival and function. Histological scores showed a similar trend: treatment with an initially low cell dose yielded significant early (2-week) benefits, whereas treatments using initial moderate or high doses performed better at later time points (4 weeks). This indicates that different initial doses confer advantages within distinct temporal windows; however, it should be noted that these findings are based on single-dose administration at the time of treatment, rather than dose adjustments during the course of healing. This phenomenon aligns with the “therapeutic window” concept proposed by Goldberg et al. (Goldberg et al., 2024). In terms of delivery strategy, scaffold-loaded stem cells outperformed direct stem cell transplantation alone in ultimate load, histological scores, and type I collagen expression. Scaffolds provide critical mechanical support and physical retention for stem cells and, through their biochemical composition and topographical features, promote cell survival, engraftment, and the synthesis and assembly of functional extracellular matrix (Du et al., 2024). For example, decellularized matrix hydrogels or aligned collagen fiber scaffolds can guide collagen deposition along the principal loading axis, thereby promoting structurally organized regeneration (Wu et al., 2024; Chen, 2025). It is noteworthy that the scaffold types included in the synergistic analysis of type I collagen expression comprised PLG scaffolds, PLGA scaffolds, collagen scaffolds, and collagen/alginate hydrogel scaffolds, which differ in material source, degradation characteristics, and microarchitecture. However, because each scaffold type was represented by only a single study, we were unable to perform scaffold-stratified subgroup analyses to explore scaffold-specific effects stem cell function. It should be emphasized that the dose- and time-related findings in this study are derived from exploratory subgroup analyses and are intended primarily to highlight potential sources of heterogeneity, rather than to provide definitive biological conclusions or dosing recommendations. Given the limited number of studies in some subgroups and the variability in cell sources, delivery methods, animal models, and testing protocols, residual heterogeneity likely remains within subgroups. Moreover, multiple subgroup comparisons were conducted without formal adjustment for multiple testing, and thus the statistical significance of these findings should be interpreted with caution. To examine robustness, we pooled the main outcomes using both fixed-effects and random-effects models, which yielded consistent effect directions and overall trends. Substantial between-study heterogeneity was mainly observed for histological scores and type I collagen outcomes, which to some extent limits the strength with which dose-related conclusions can be generalized.

The results of this study are consistent with most preclinical reports, collectively supporting the positive efficacy of stem cells in Achilles tendon repair. For instance, Liu et al. and Yu et al. both reported that stem cell transplantation can upregulate the expression of tendon-related genes and improve biomechanical performance (Yu et al., 2020; Liu et al., 2017). Chamberlain et al. also found that stem cells could inhibit excessive deposition of type III collagen and promote physiological remodeling of type I collagen (Chamberlain et al., 2017). In terms of combined scaffolds, the conclusions of this study align with trends in the field of tissue engineering, as Cai et al. confirmed that polyurethane scaffolds loaded with BMSC significantly enhanced ultimate load and collagen content (Cai et al., 2018). However, some studies differ from our results; for example, Guo et al. reported that stem cells significantly improved stiffness, while this meta-analysis did not reach the same conclusion (Guo et al., 2020). Such discrepancies may arise from differences in models or inconsistencies in experimental conditions, such as mechanical testing methods. Additionally, Bowers’ study suggested that regardless of stem cell dosage, there were no significant differences in histological scores or type I collagen deposition between the stem cell treatment and control groups (Bowers et al., 2022). In contrast, our animal data indicate that the therapeutic effects of stem cells exhibit a more complex dose-effect relationship, potentially influenced by species specificity and local microenvironmental factors.

### 4.1 Research limitations and prospects for clinical translation

This study has several limitations. First, its conclusions must be interpreted with caution in light of the methodological quality of the included studies. According to the SYRCLE assessment, most studies were rated as “unclear” risk in key domains, including random sequence generation, allocation concealment, blinding of interventions, blinding of outcome assessment, and selective reporting, primarily due to insufficient reporting. In animal experiments, inadequate implementation or reporting of randomization and blinding can lead to overestimation of effect sizes; thus, the magnitude of the effects observed in this meta-analysis may be influenced by these issues. It should be emphasized that performance and detection bias are largely determined by the original experimental design and conduct, and cannot be eliminated by statistical techniques in a meta-analysis. Analyses of publication bias and trim-and-fill procedures address only selective publication and cannot substitute for control of performance or measurement bias. On this basis, the pooled effects in the present study are best viewed as a quantitative summary of overall trends. Future studies should strengthen preregistration, randomization and allocation concealment, blinding, and the prespecification and complete reporting of primary outcomes. Second, although scaffold-loaded stem groups showed larger effects in this study, the current evidence does not allow clear separation of the contribution of stem cells from the mechanical support or biological effects of the scaffold itself. The scaffolds used differed markedly in material composition and mechanical properties, and their characterization and reporting were inconsistent across studies, limiting the feasibility and interpretability of further stratification by specific scaffold type. In the synergistic analysis of type I collagen expression, for example, each of the four scaffold types (PLG, PLGA, collagen, and collagen/alginate hydrogel) was represented by only a single study, precluding evaluation of scaffold-specific effects on stem cell function. We therefore reported the comparisons of scaffold-loaded stem cells versus scaffold alone, and of stem cells versus negative controls, to describe both the relative gain of composite strategies and the overall stem cell–associated effect. However, under conditions of substantial scaffold heterogeneity, the independent contribution of stem cells cannot be estimated precisely. Future work should adopt standardized scaffold characterization and control conditions, and conduct more consistently designed comparative studies to strengthen the evidence for stem cell–specific effects. Similarly, we combined stem cells from different sources (BMSCs, ADMSCs, TDSCs,.) in the same pooled analyses, with the aim of estimating the overall effect of “stem cell–based interventions”, rather than implying functional equivalence. Stem cells from different sources differ substantially in their biological characteristics, tenogenic differentiation potential, and paracrine profiles, and their therapeutic effects should not be expected to be identical (Yuan et al., 2023). Because stratification by cell source resulted in very small sample sizes in most subgroups, stable pooled estimates could not be obtained, and we did not perform cell source–based subgroup meta-analyses. The overall trends were consistent under both fixed-effects and random-effects models, suggesting a degree of statistical robustness, but this does not justify the inference that all cell sources have similar efficacy. More standardized studies and head-to-head comparisons are urgently needed to clarify how cell source influences therapeutic outcomes.

At the molecular level, we pooled gene and protein expression data for type I collagen. Although this approach was necessary to maintain statistical power for subgroup analyses, it may obscure differences between transcription and translation and is one of the central limitations of this study. We have discussed this issue in detail and stressed that the pooled effect sizes should be interpreted as a statistical summary of overall differences in type I collagen expression, rather direct evidence of collagen structural maturation. In addition, the analysis of type I collagen was subject to notable temporal bias: most included studies assessed outcomes at 2–4 weeks postoperatively, which corresponds to the early phase of the transition from type III to type I collagen during tendon healing. This may have led to underestimation of the true effect of stem cells on promoting type I collagen deposition. For biomechanical outcomes, there was substantial heterogeneity in stiffness measurement methods. Although most studies defined stiffness as the slope of the linear region of the load–displacement curve, some used alternative calculation approaches. This methodological inconsistency may have increased between-study heterogeneity and complicates interpretation of the pooled results. Furthermore, while there was evidence of potential publication bias for some primary outcomes, trim-and-fill correction did not materially alter the conclusions, which to some extent strengthens confidence in the findings. Finally, it is important to note that all evidence synthesized in this study derives from rat models with predominantly short-term follow-up, reflecting early phases of healing. Given the pronounced differences between rats and humans in loading patterns, mechanical environment, and immune responses, the conclusions of this meta-analysis should be confined to trends in structural repair at animal level and should not be directly extrapolated to long-term functional recovery in clinical settings (Goldberg et al., 2024).

Looking ahead, the clinical translation of stem cell therapy for Achilles tendon injuries still faces multiple challenges. Central issues include how to further enhance post-transplant cell survival and the durability of their function, which may be optimized through strategies such as engineering hydrogel encapsulation, co-delivery of pro-survival factors, or gene editing (e.g., overexpressing anti-apoptotic or tenogenic genes) (Ning et al., 2023). Although long-term safety concerns—including immune rejection, ectopic ossification, and the theoretical risk of tumorigenesis—have been rarely observed in current animal models, they must be systematically evaluated in large-animal studies with extended follow-up before clinical application (Zhuang et al., 2021; Taechangam et al., 2022). In addition, combining gene therapy with stem cell therapy may represent an important future direction. Sherwin et al. (Sherwin et al., 2023) recently demonstrated that the AAV2.5 serotype can efficiently transduce tendon cells and sustain gene expression for 7 days, offering a potential platform for local delivery of growth factors or modulation of key signaling pathways. When used in combination with stem cell transplantation, gene therapy could enhance the anti-apoptotic, pro-angiogenic, or lineage-specific differentiation capacities of stem cells, overcoming some limitations of cell therapy alone. In a bibliometric analysis, Zhang et al. (Zhang et al., 2024) identified scaffolds, molecular mechanisms, and inflammation regulation as the three major hotspots in current TDSC research, which is highly consistent with the trends reflected by our findings. Accordingly, future studies should aim to establish standardized protocols for stem cell preparation and delivery to reduce heterogeneity (Petrus-Reurer et al., 2021); integrate multi-omics approaches (such as single-cell transcriptomics) to elucidate *in vivo* cell fate and paracrine mechanisms; and explore combination strategies that pair stem cells with growth factors (e.g., GDF-5, CTGF) or physical stimuli (e.g., mechanical loading). Crucially, when reporting molecular outcomes such as type I collagen, investigators should clearly distinguish between gene and protein expression levels and, whenever possible, report both to enable more refined meta-analyses and deeper investigation of how stem cell therapy differentially affects transcription and translation. In the biomechanical domain, there is an urgent need to standardize stiffness measurement and reporting, with explicit definitions of the stiffness type used (e.g., linear stiffness, secant stiffness) and the segment of the load–displacement curve on which calculations are based in order to reduce methodological heterogeneity and improve comparability across studies. For scaffold materials, future research should prioritize head-to-head comparative studies of different scaffolds to determine which material properties are most conducive to stem cell–mediated tendon regeneration. Overall, upcoming work should focus on standardized cell preparation and delivery, extended follow-up, and more comprehensive mechanical and functional assessment in large-animal models to verify long-term efficacy and safety, and only then cautiously advance to early clinical trials.

## 5 Conclusion

Stem cell transplantation, particularly when combined with biomimetic scaffolds, can markedly enhance tensile strength and histological remodeling after Achilles tendon rupture, indicating a clear benefit for structural tendon repair. However, the current evidence is still insufficient to support the achievement of full functional regeneration. Although ultimate load and histological scores improve significantly, key functional mechanical parameters such as stiffness do not show consistent enhancement at most time points, suggesting that the mechanical performance of the repair tissue has not fully returned to that of normal tendon. With respect to type I collagen expression, the effect of stem cell therapy alone appears limited, a finding that may be partly influenced by the relatively early evaluation time points. Most studies assessed outcomes at 2–4 weeks postoperatively, during the early phase of collagen transition, which may underestimate the long-term impact of stem cells on collagen maturation. It should be emphasized that the conclusions of this study reflect overall trends derived from pooled analyses across multiple stem cell sources and scaffold materials. Given the inherent biological differences among stem cells from different sources, and the distinct physicochemical properties and biological functions of various scaffolds, the optimal cell–scaffold combination cannot yet be defined. This study further indicates delivery method, transplantation dose, and follow-up duration are major contributors to heterogeneity in treatment efficacy. Critically, we found that most current preclinical studies provide inadequate reporting in key methodological domains such as randomization and blinding, leading to a potential risk of bias and highlighting that the evidentiary foundation in this field remains fragile. Overall, combining stem cells with biomimetic scaffolds appears to enhance structural repair and load-bearing capacity, but evidence for long-term recovery of functional biomechanics remains limited. High-quality, rigorously designed studies with longer follow-up, standardized mechanical testing, and validation in large-animal models are urgently needed to substantiate these findings.

## Data Availability

The original contributions presented in the study are included in the article/Supplementary Material, further inquiries can be directed to the corresponding author.
