# Supplementary material for: Efficacy of stem cell therapy for achilles tendon rupture: a systematic review and meta-analysis based on animal studies
Source: Front Bioeng Biotechnol. 2026 Jun 1;14:1753018. doi: 10.3389/fbioe.2026.1753018 (PMC13265556; doi:10.3389/fbioe.2026.1753018)
Supplement: Supplementary file 1 [file Supplementaryfile1.docx]

**Efficacy of Stem Cell Therapy for Achilles Tendon Rupture: A Systematic Review and Meta-Analysis Based on Animal Studies**

**Table 1: Search strategies**

| **PubMed**  #1: "Stem Cells"[MeSH Terms]  #2: "stem cell"[Title/Abstract] OR "stem cells"[Title/Abstract] OR "mesenchymal stem cells"[Title/Abstract] OR "MSC"[Title/Abstract] OR "adipose-derived stem cells"[Title/Abstract] OR "ADSC"[Title/Abstract] OR "bone marrow stem cells"[Title/Abstract] OR "umbilical cord mesenchymal stem cells"[Title/Abstract] OR "hUC-MSC"[Title/Abstract]  #3: #1 OR #2  #4: "achilles Tendon"[MeSH Major Topic]  #5: "achilles tendon injury"[Title/Abstract] OR "achilles tendon rupture"[Title/Abstract] OR "achilles tendon defect"[Title/Abstract] OR "tendon injury"[Title/Abstract] OR "achilles tendon"[Title/Abstract]  #6: #4 OR #5  #7: #3 AND #6  **Web of science**  (TS=(“stem cell” OR “stem cells” OR “mesenchymal stem cells” OR “MSC” OR “adipose-derived stem cells” OR “ADSC” OR “bone marrow stem cells” OR “umbilical cord mesenchymal stem cells” OR “hUC-MSC”)) AND TS=(“achilles tendon injury” OR “achilles tendon rupture” OR “achilles tendon defect” OR “tendon injury” OR “achilles tendon”)  **Embase**  #1: 'stem cell':ti,ab,kw OR 'stem cells':ti,ab,kw OR 'mesenchymal stem cells':ti,ab,kw OR 'msc':ti,ab,kw OR 'adipose-derived stem cells':ti,ab,kw OR 'adsc':ti,ab,kw OR 'bone marrow stem cells':ti,ab,kw OR 'umbilical cord mesenchymal stem cells':ti,ab,kw OR 'huc-msc':ti,ab,kw  #2: 'stem cell'/exp  #3: 'stem cells'/exp  #4: 'mesenchymal stem cells'/exp  #5: 'adipose-derived stem cells'/exp  #6: 'umbilical cord mesenchymal stem cells'/exp  #7: #1 OR #2 OR #3 OR #4 OR #5 OR #6  #8: 'achilles tendon injury':ti,ab,kw OR 'achilles tendon rupture':ti,ab,kw OR 'achilles tendon defect':ti,ab,kw OR 'tendon injury':ti,ab,kw OR 'achilles tendon':ti,ab,kw  #9: 'achilles tendon injury'/exp  #10: 'achilles tendon rupture'/exp  #11: 'tendon injury'/exp  #12: 'achilles tendon'/exp  #13: #8 OR #9 OR #10 OR #11 OR #12  #14: #7 AND #13  #15: #14 AND ('article'/it OR 'review'/it)  **Scopus**  TITLE-ABS-KEY ( "stem cell" OR "stem cells" OR "mesenchymal stem cells" OR "MSC" OR "adipose-derived stem cells" OR "ADSC" OR "bone marrow stem cells" OR "umbilical cord mesenchymal stem cells" OR "hUC-MSC" ) AND TITLE-ABS-KEY ( "achilles tendon injury" OR "achilles tendon rupture" OR "achilles tendon defect" OR "tendon injury" OR "achilles tendon" ) AND ( LIMIT-TO ( DOCTYPE , "ar" ) OR LIMIT-TO ( DOCTYPE , "re" ) ) |
| --- |

**Table 2: Basic information of included studies**

| **No.** | **Author** | **Year** | **Country** | **Study Type** | **Baseline Characteristics** | | | | **Model Type** | **Specific Method** | **Stem Cells** | | |  | **Sample Size** | **Graft** | **Follow-up Endpoint** |
| --- | --- | --- | --- | --- | --- | --- | --- | --- | --- | --- | --- | --- | --- | --- | --- | --- | --- |
|  |  |  |  |  | **Species** | **Sex** | **Weight** | **Age** |  |  | **Type** | **Source** | **Dose** | **Dose Grouping** |  |  |  |
| 1 | UYAR | 2022 | Turkey | RCT | Wistar Rat | Male | 300-350g | 8-12 weeks | Achilles tendon rupture | The left Achilles tendon of the rat was severed. | ADMSCs | Human subcutaneous fat | / | / | 10/10 | / | 8 weeks |
| 2 | Später | 2024 | USA | RCT | nude rat | Female | 227.5 ± 19.6g | 18 weeks | Achilles tendon rupture | A 3mm section was excised from the middle region of the Achilles tendon. | iMSCs | Human-induced pluripotent stem cell (iPSC)-derived mesenchymal stem cells (iMSCs) | 0.5×10^6 | low dose | 12/12 | Collagen scaffold | 9 weeks |
| 3 | Aktas | 2016 | Turkey | RCT | Wistar Rat | Male | / | / | Achilles tendon rupture | A 3mm segmental defect was created in the Achilles tendon. | BMSCs | Rat bone marrow | 1×10^6 | middle dose | 24/24 | Poly(lactic-co-glycolic acid) (PLG) scaffold | 4 weeks |
| 4 | Öztürk | 2023 | Turkey | RCT | Wistar Rat | Female | 300-350g | / | Achilles tendon rupture | A full-thickness window defect with a diameter of 1mm was made in the Achilles tendon using a punch. | hUCMSCs | Human umbilical cord | 0.68×10^6 | low dose | 16/16 | / | 4 weeks |
| 5 | Pietschmann | 2012 | Germany | RCT | Lewis Rat | Female | 190 ± 15g | 7 weeks | Achilles tendon rupture | A 3mm section from the middle of the Achilles tendon was excised. | BMSCs | Rat bone marrow | 1×10^6 | middle dose | 11/11 | Collagen scaffold | 16 weeks |
| 6 | Zhang | 2018 | China | RCT | SD Rat | Female | 250±10g | 8 weeks | Achilles tendon rupture | A portion of the right hind limb Achilles tendon was excised, creating a defect approximately 5mm long and 1mm wide. | BMSCs | Rat bone marrow | / |  | 6/6/6 | Collagen scaffold | 4 weeks |
| 7 | Zhao | 2019 | China | RCT | SD Rat | Female | 250±20g | 6-8 weeks | Achilles tendon rupture | A defect 7mm in length was created. | BMSCs | Rat bone marrow | / |  | 30/30 | Poly(lactic-co-glycolic acid) (PLGA) scaffold | 8 weeks |
| 8 | Yuksel | 2016 | Turkey | RCT | SD Rat | Male | 400-500g | / | Achilles tendon rupture | A transverse incision was made 4-5mm proximally at the tendon-bone junction of the Achilles tendon. | BMSCs | Rat bone marrow | 1×10^6 | middle dose | 10/10 | / | 4 weeks |
| 9 | D. Lucke | 2018 | Brazil | RCT | Wistar Rat | Male | / | 15 weeks | Achilles tendon rupture | A partial transverse incision was made 4mm from the attachment point of the calcaneus, in the proximal tendon region (approximately 2/3 of the injury depth). | ADMSCs | Fat tissue from the inguinal region of rats | 0.45×10^6 | low dose | 16/16 | / | 2 weeks |
| 10 | Al-ani | 2015 | China | RCT | SD Rat | Male | 200g | / | Achilles tendon rupture | The middle portion of the Achilles tendon was excised 5mm from the attachment point of the calcaneus. The incision was sutured using clinical sutures. | TDSCs\BMSCs | Rat Achilles tendon and bone marrow | 1×10^6 | middle dose | 24/24/24 | / | 4 weeks |
| 11 | Guo | 2019 | China | RCT | SD Rat | Female | 250-300g | / | Achilles tendon rupture | The Achilles tendon was severed, creating a 3mm gap in the tendon. | TDSCs | Bilateral Achilles tendon tissue from a miscarriage fetus | 10×10^6 | high dose | 12/12 | / | 8 weeks |
| 12 | Lee | 2017 | South Korea | RCT | SD Rat | Male | 390-410g | 13 weeks | Achilles tendon rupture | A standard parallel incision 5mm long and spaced 0.8mm apart was made on the tendon. | ADMSCs | Human subcutaneous fat | 1×10^6 | middle dose | 18/18 | Collagen scaffold | 4 weeks |
| 13 | B. Adams | 2014 | USA | RCT | SD Rat | Male | 370±34g | 96±22d | Achilles tendon rupture | A 3mm long section of the Achilles tendon was removed. | BMSCs | Human iliac crest bone marrow | 1×10^6 | middle dose | 12/12 | / | 4 weeks |
| 14 | Aynardi | 2018 | USA | RCT | SD Rat | Not limited | / | 12 weeks | Achilles tendon rupture | The Achilles tendon was excised from the bony attachment point to 1mm distal to the tendon-muscle junction. | ADMSCs | Fat tissue from the inguinal region of rats | 0.1×10^6 | low dose | 10/10 | / | 4 weeks |
| 15 | O. Bortolazzo | 2020 | Brazil | RCT | Wistar Rat | Male | / | 15 weeks | Achilles tendon rupture | A partial transverse incision was made in the proximal tendon region about 4mm from the calcaneal attachment, covering approximately two-thirds of the injury depth. | ADMSCs | Fat tissue from the inguinal region of rats | 0.45×10^6 | low dose | 17/17 | / | 2 weeks |
| 16 | Norelli | 2018 | USA | RCT | SD Rat | Male | 350-400g | 12 weeks | Achilles tendon rupture | A cylindrical defect with a diameter of 2mm was created in the middle of the tendon using a surgical tendon punch. | ADMSCs | Rat inguinal fat pad | 3×10^6 | high dose | 45/45 | Scaffold made of a mixture of collagen and alginate gel | 4.5 weeks |
| 17 | Schon | 2014 | USA | RCT | SD Rat | Male | 250-300g | / | Achilles tendon rupture | The Achilles tendon was completely severed at the junction between the Achilles tendon and the gastrocnemius/soleus muscles. The muscle was transected 3mm from the tendon end to create a defect between the tendon and muscle tissue. | BMSCs | Human bone marrow | 0.4×10^6 | low dose | 15/15 | Collagen/alginate gel scaffold | 2 weeks |
| 18 | Oliva | 2019 | Italy | RCT | Lewis Rat | Male | / | 6-8 weeks | Achilles tendon rupture | A 2mm diameter defect was created in the Achilles tendon. | BMSCs | Tibia and femur of Lewis rats | 4×10^6 | high dose | 3/3 | / | 4 weeks |
| 19 | Nourissat | 2010 | France | RCT | Wistar Rat | Male | 300g | 12 weeks | Achilles tendon rupture | The Achilles tendon was severed at its bony insertion, and the attachment site was damaged by a drill. | BMSCs | Tibia and femur of Wistar rats | 4×10^6 | high dose | 39/42 | / | 6 weeks |
| 20 | Okamoto | 2010 | Japan | RCT | Fisher Rat | Male | 220-240g | 13 weeks | Achilles tendon rupture | A complete transverse incision was made 7mm above the calcaneal attachment of each Achilles tendon using a scalpel. | BMSCs | Tibia and femur of Fisher rats | 3×10^6 | high dose | 29/29 | / | 5 weeks |
| 21 | Mao | 2022 | China | RCT | SD Rat | Male | 200-250g | 8 weeks | Achilles tendon rupture | A defect was made in the Achilles tendon using a 3mm diameter perforator. | TDSCs | Rat Achilles tendon | 1×10^6 | middle dose | 12/12 | Collagen scaffold | 12 weeks |
| 22 | SELEK | 2014 | Turkey | RCT | Wistar Rat | Female | 200-300g | / | Achilles tendon rupture | A transverse incision was made 0.5cm from the proximal calcaneal attachment point of the Achilles tendon. | BMSCs | Tibia and femur of Wistar rats | 1×10^6 | middle dose | 17/17 | / | 8 weeks |
| 23 | Guo | 2021 | China | RCT | SD Rat | Male | 250-300g | 8-10 weeks | Achilles tendon rupture | A 5mm section of tissue was excised from the middle of the Achilles tendon. | ADMSCs | Inguinal fat from SD rats | 1.5×10^6 | high dose | 20/20 | Collagen scaffold | 4 weeks |
| 24 | Zhang | 2024 | China | RCT | SD Rat | Not limited | 200-220g | / | Achilles tendon rupture | A transverse section of the Achilles tendon was exposed approximately 5mm above the calcaneus. | ADMSCs | Inguinal fat from SD rats | 3×10^6 | high dose | 12/12 | / | 8 weeks |
| 25 | Yang | 2020 | China | RCT | SD Rat | Male | 200-220g | / | Achilles tendon rupture | A complete transverse incision was made 7mm above the calcaneal attachment point. | ADMSCs | Rat inguinal fat pad | 0.1×10^6 | low dose | 16/16 | Polymethyl methacrylate (PMMA) scaffold | 4 weeks |
| 26 | Mao | 2025 | China | RCT | SD Rat | / | 350±50g | 8-10 weeks | Achilles tendon rupture | The Achilles tendon was severed 5mm above the calcaneus. | TDSCs | Rat Achilles tendon | / |  | 24/24 | Decellularized amniotic membrane scaffold | 12 weeks |
| 27 | Wang | 2023 | China | RCT | SD Rat | Male | 200-250g | / | Achilles tendon rupture | The Achilles tendon was transected 5mm from the calcaneal insertion point. | TDSCs | Rat Achilles tendon | 1×10^6 | middle dose | 30/30 | / | 3 weeks |

**
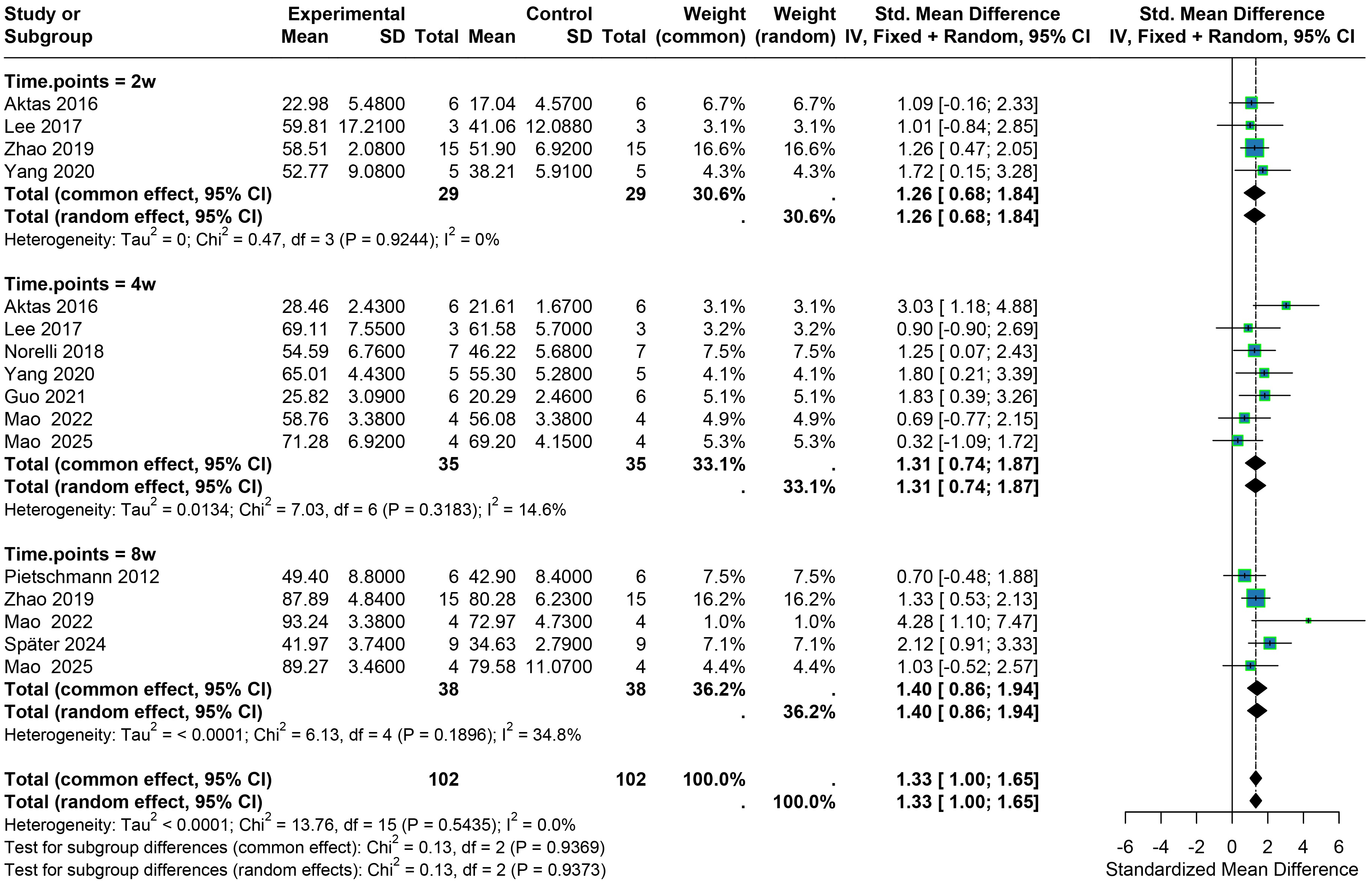
**

**Supplementary Figure 1: Maximum load comparison between Scaffold + Stem Cells and Scaffold groups.**

**
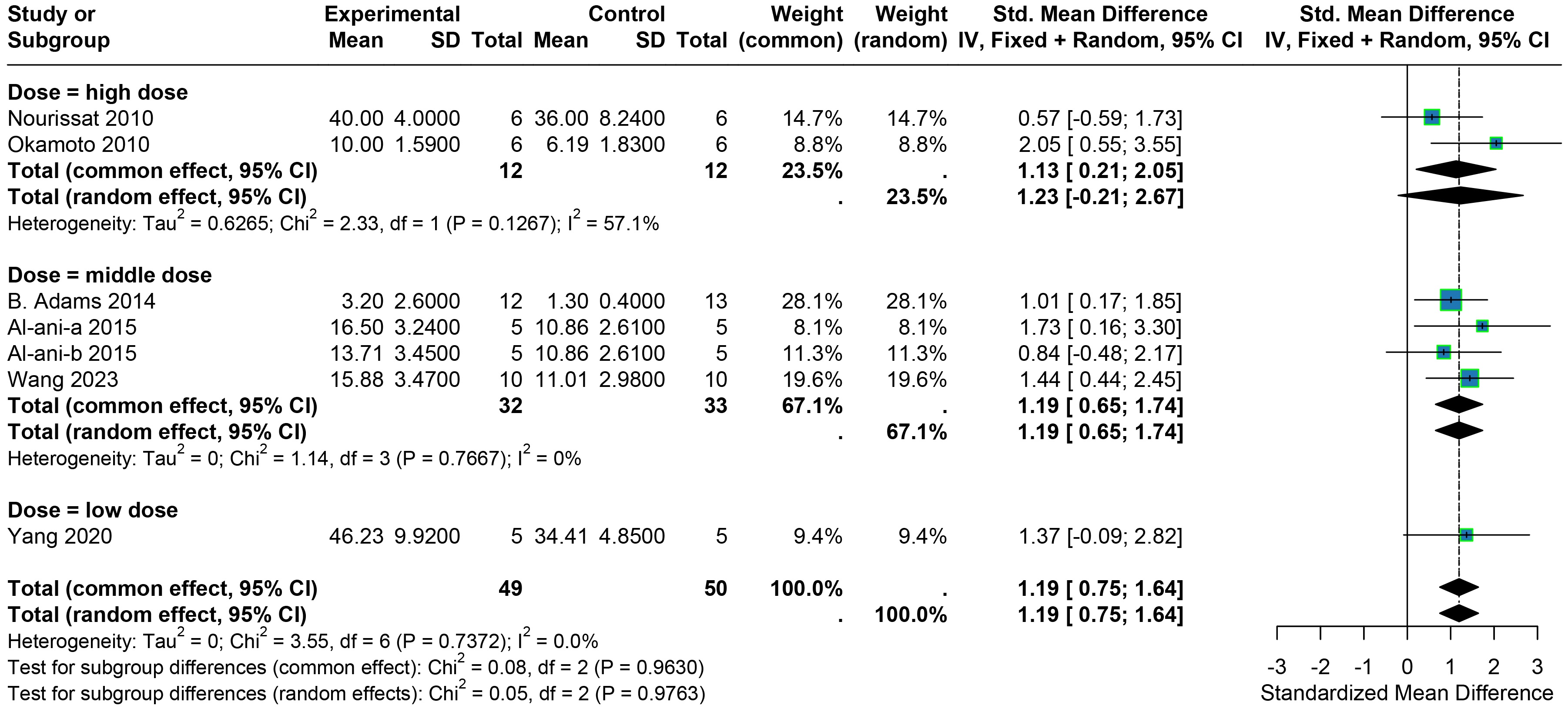
**

**Supplementary Figure 2: Maximum load comparison between stem cells and control groups at 2 weeks - dosage subgroup.**

**
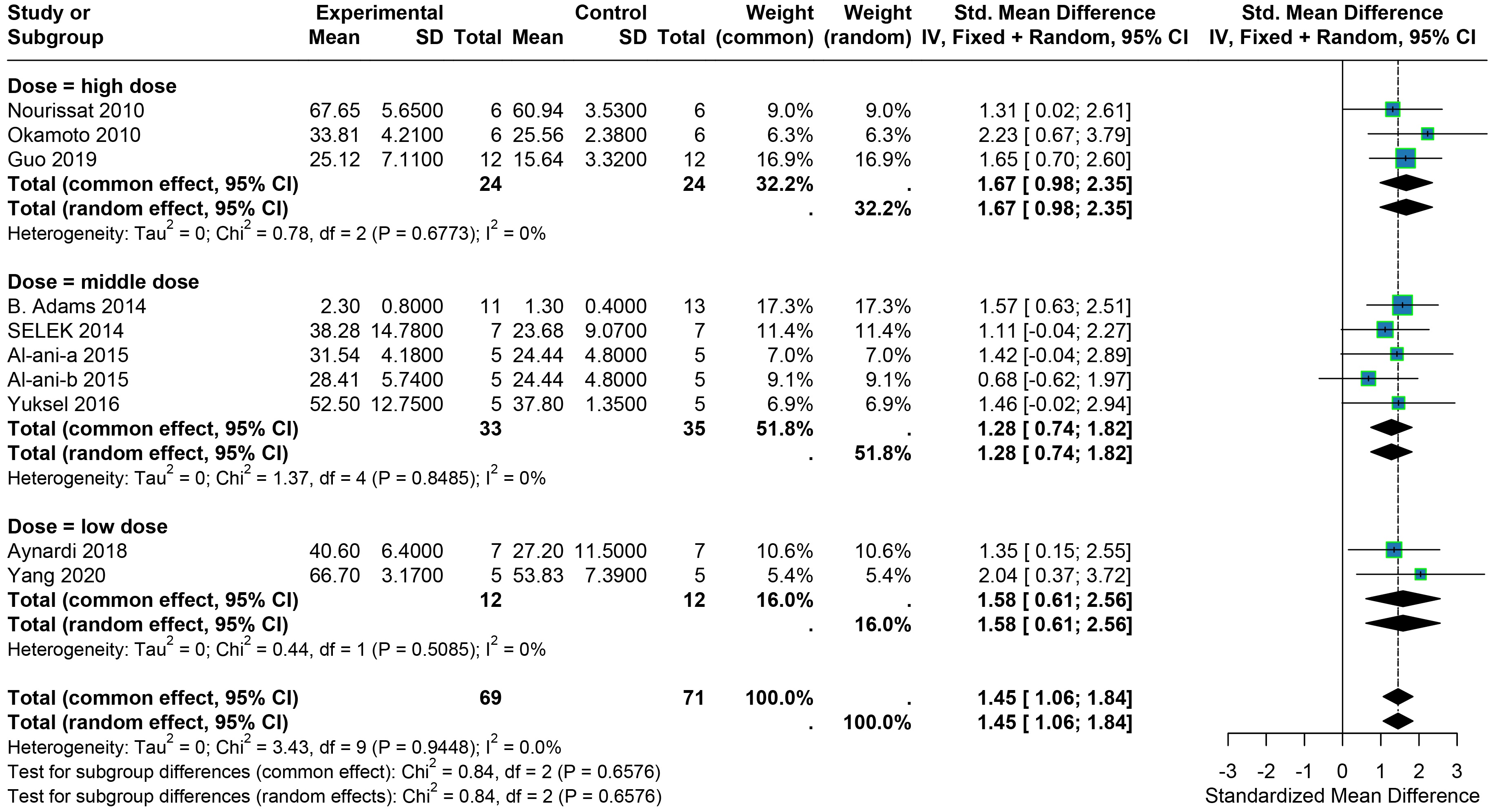
**

**Supplementary Figure 3: Maximum load comparison between stem cells and control groups at 4 weeks - dosage subgroup.**

**
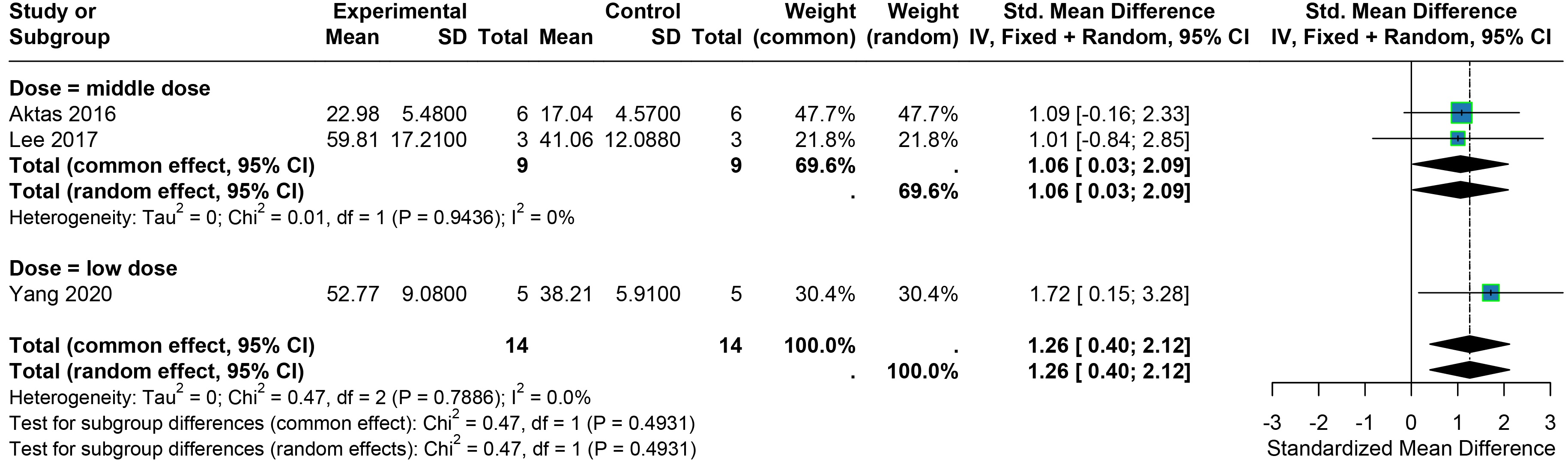
**

**Supplementary Figure 4: Maximum load comparison between Scaffold + Stem Cells and Scaffold groups at 2 weeks - dosage subgroup.**

**
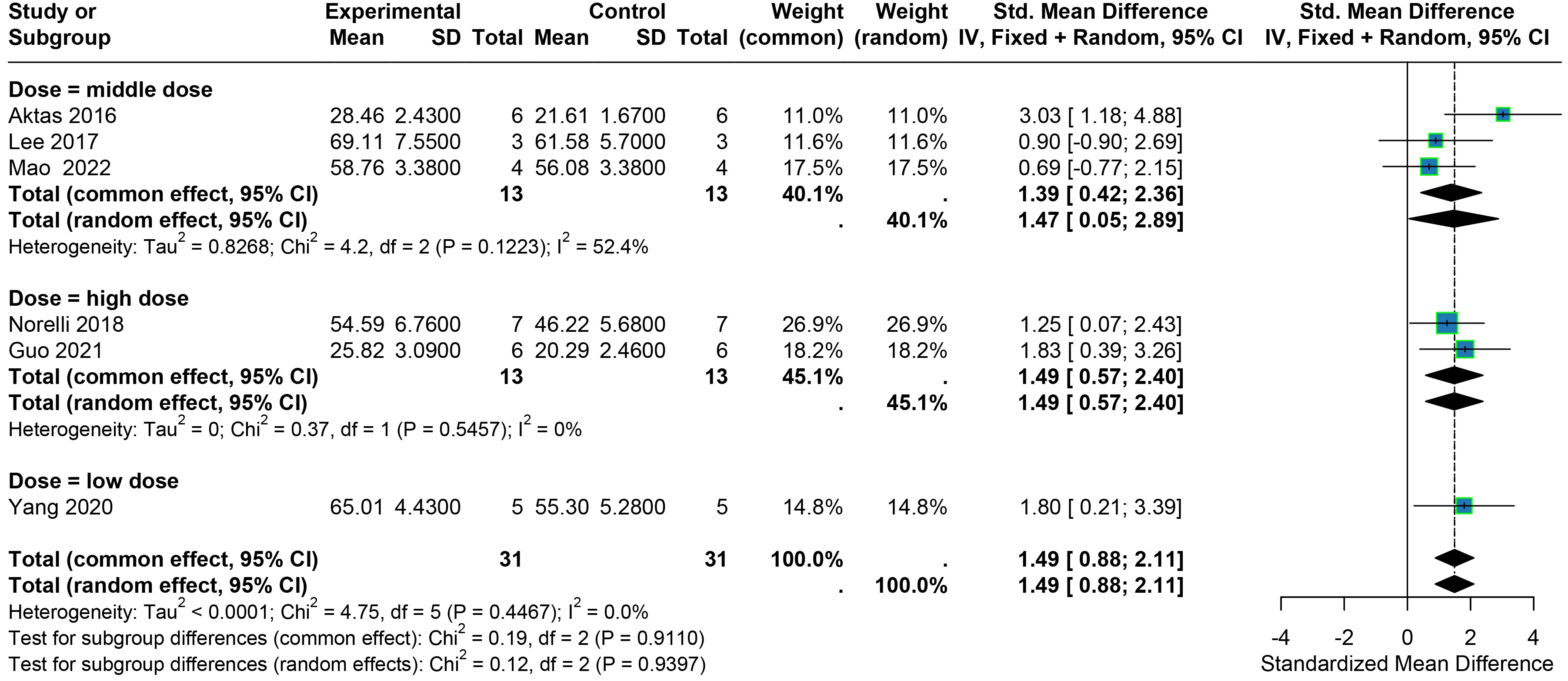
**

**Supplementary Figure 5: Maximum load comparison between Scaffold + Stem Cells and Scaffold groups at 4 weeks - dosage subgroup.**

**
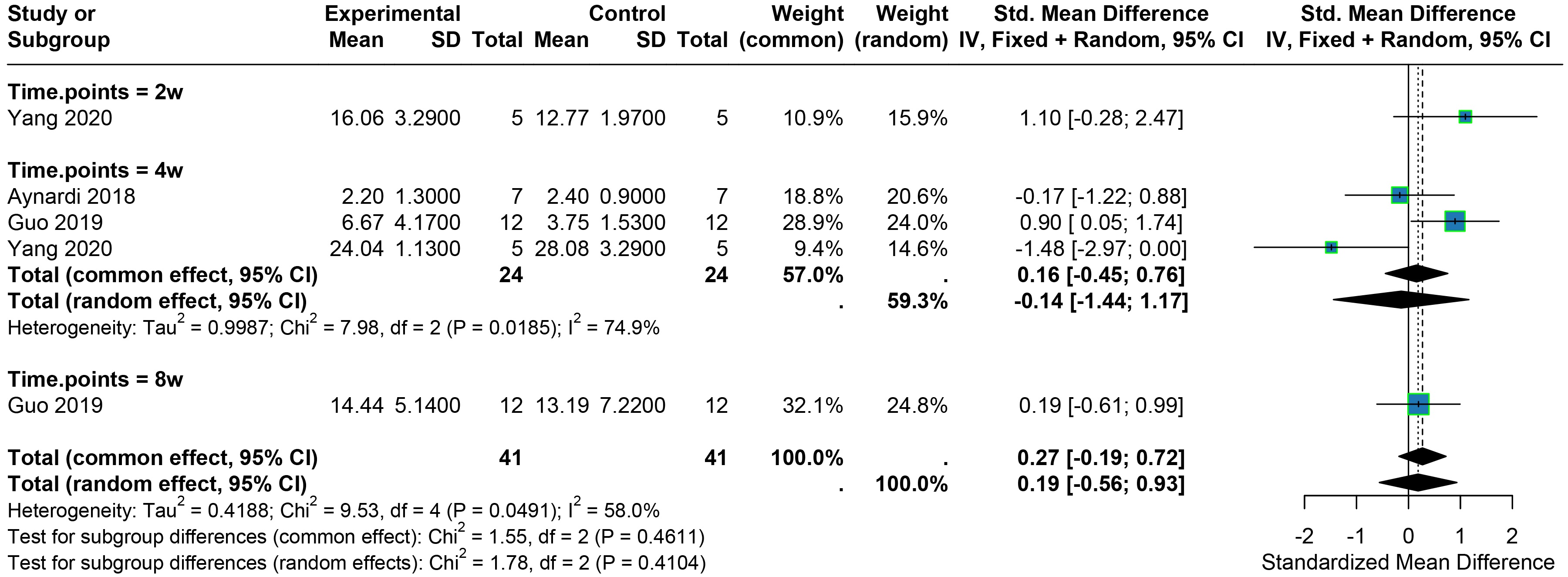
**

**Supplementary Figure 6: Stiffness comparison between stem cells and control groups.**

**
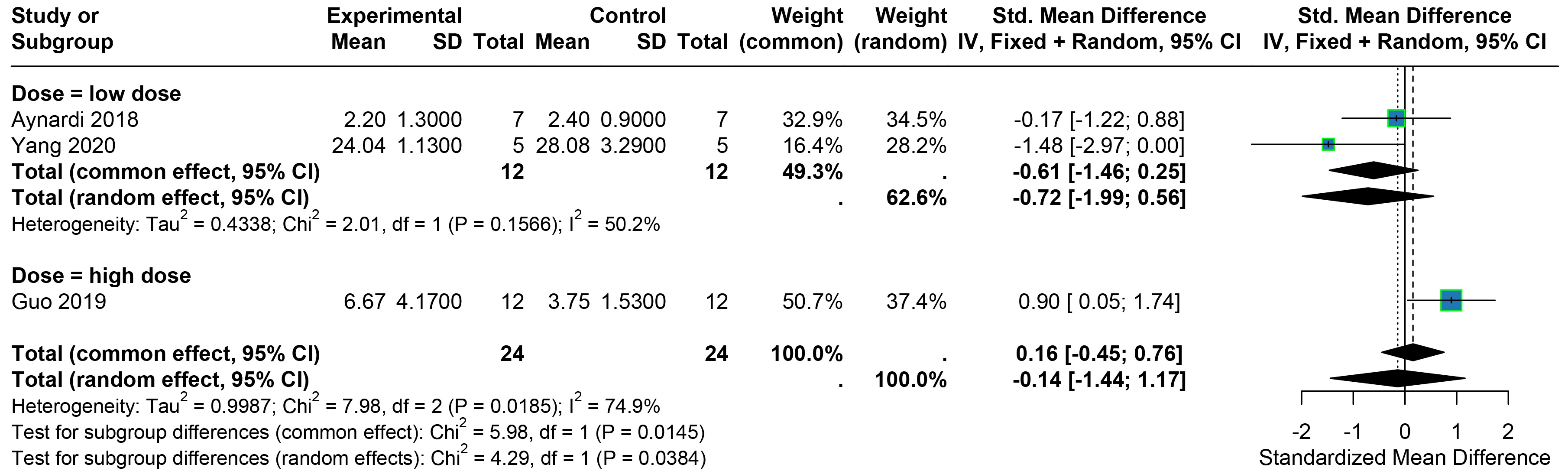
**

**Supplementary Figure 7: Stiffness comparison between stem cells and control groups at 4 weeks - dosage subgroup.**

**
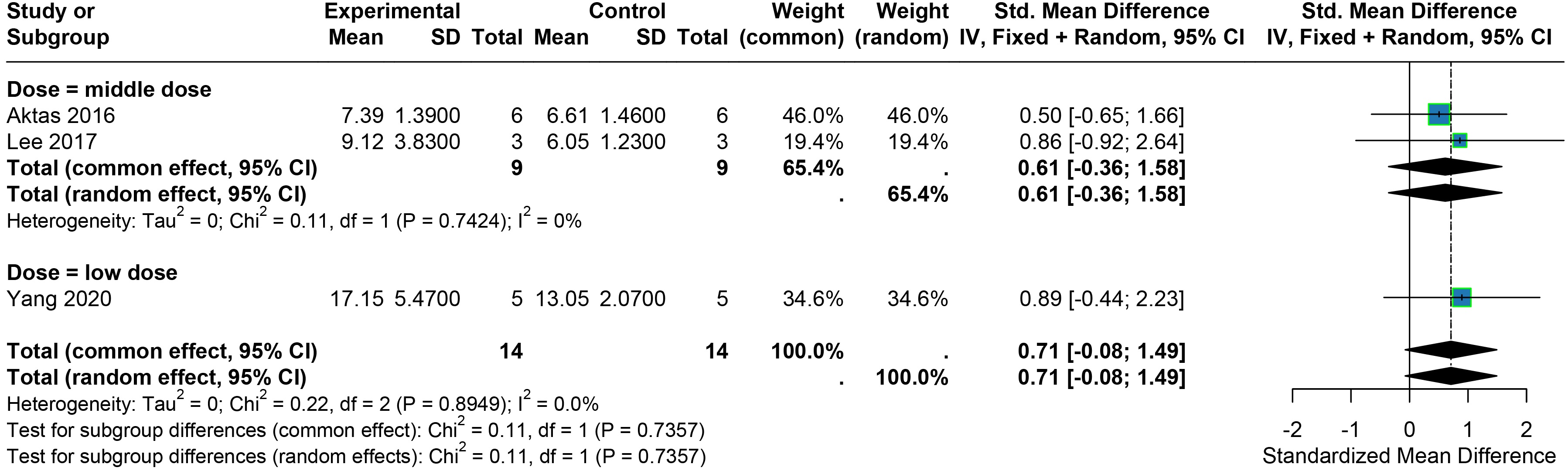
**

**Supplementary Figure 8: Stiffness comparison between Scaffold + Stem Cells and Scaffold groups at 2 weeks - dosage subgroup.**

**
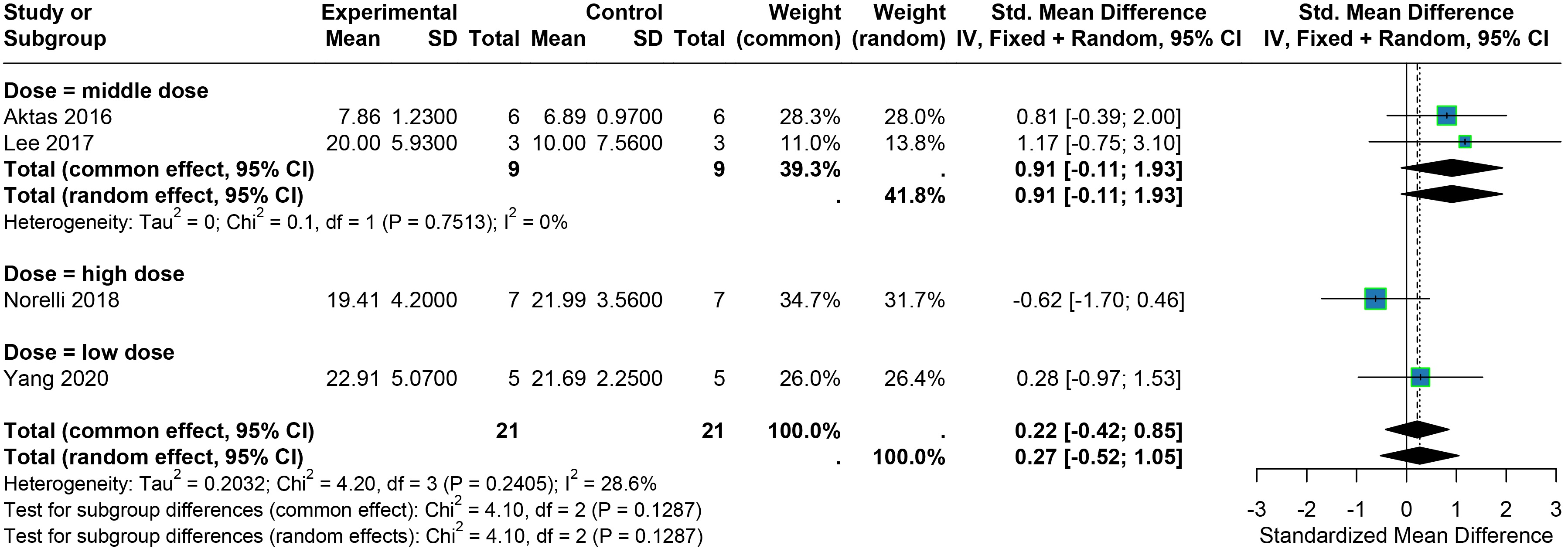
**

**Supplementary Figure 9: Stiffness comparison between Scaffold + Stem Cells and Scaffold groups at 4 weeks - dosage subgroup.**

**
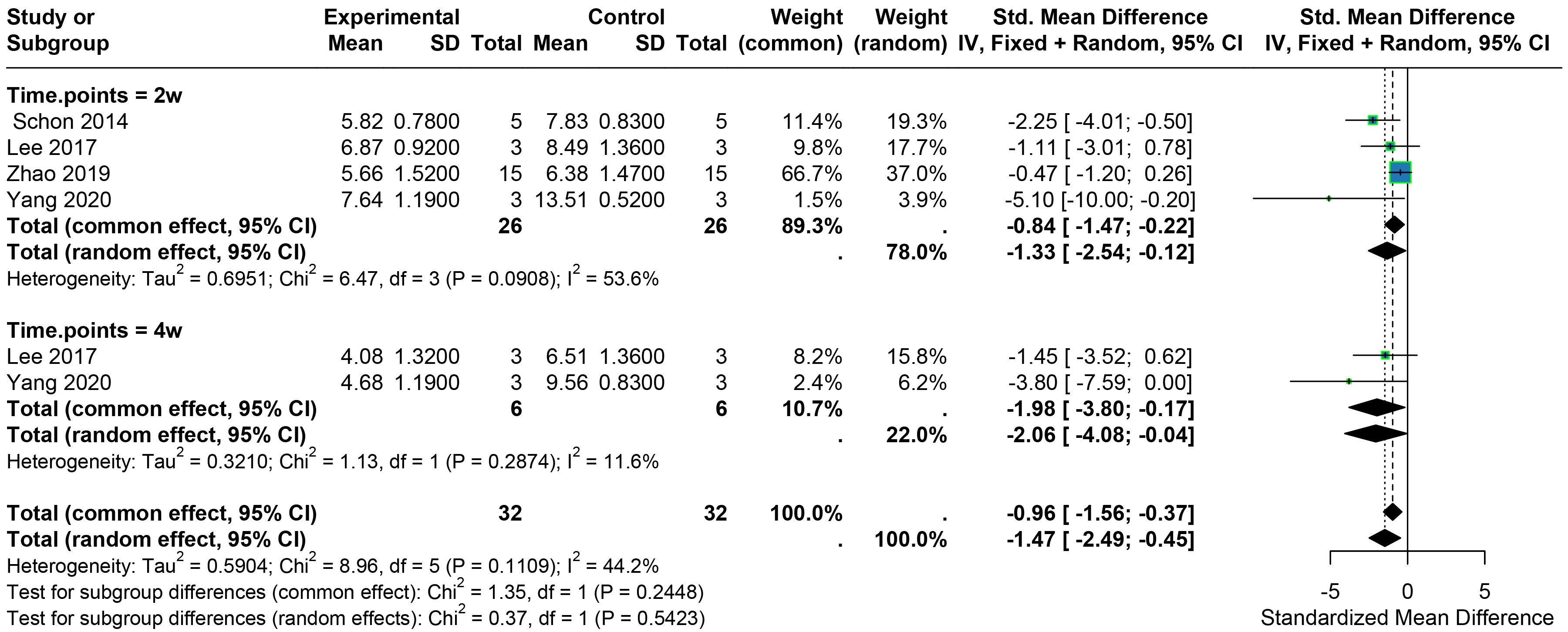
**

**Supplementary Figure 10: Histological scores comparison between Scaffold + Stem Cells and Scaffold groups.**

**
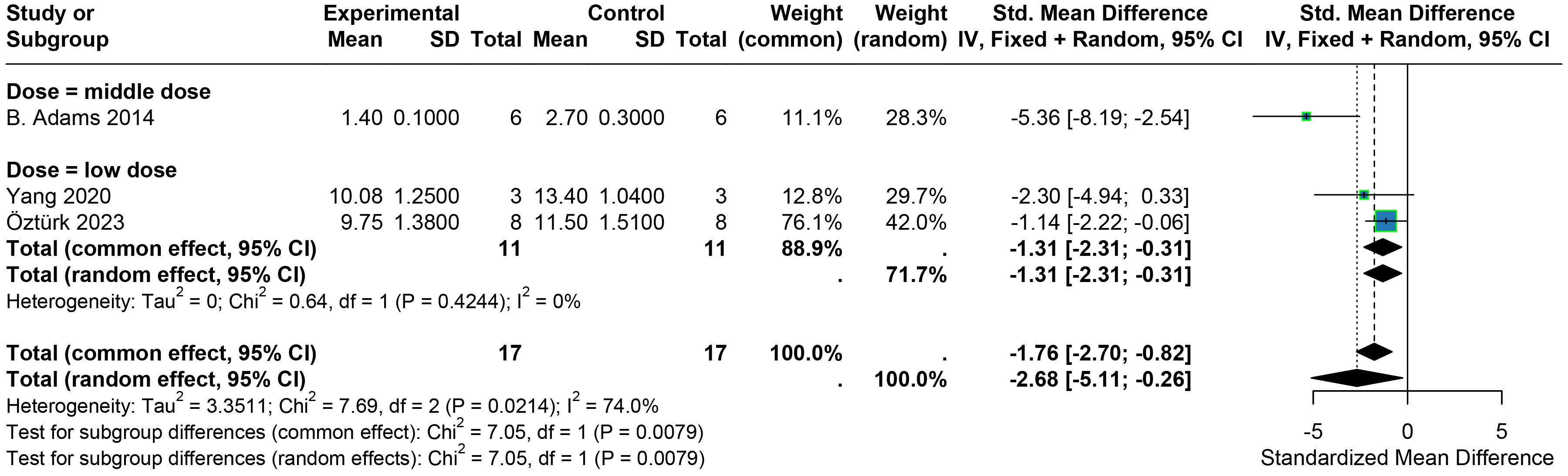
**

**Supplementary Figure 11: Histological scores comparison between stem cells and control groups at 2 weeks - dosage subgroup.**

**
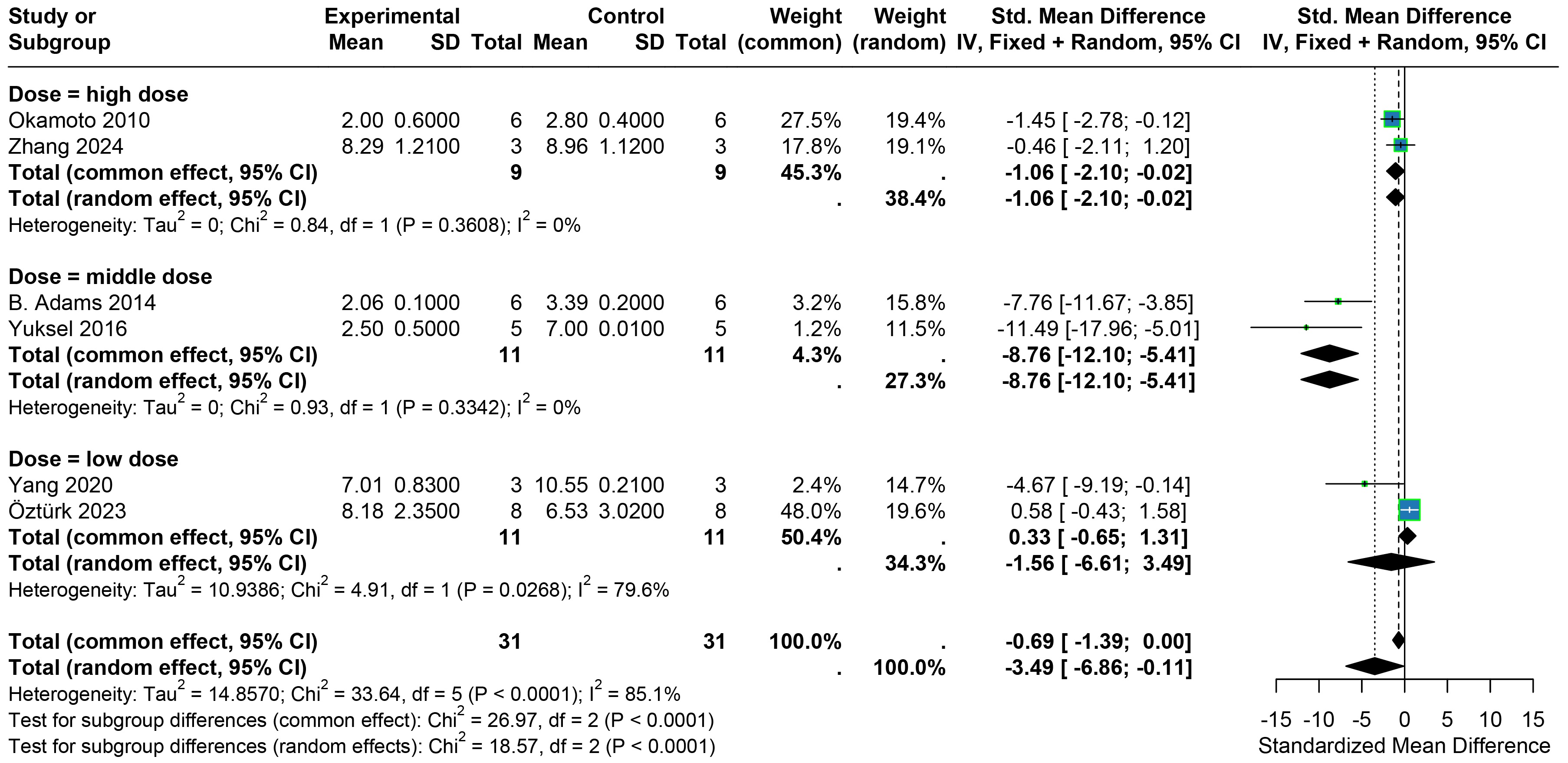
**

**Supplementary Figure 12: Histological scores comparison between stem cells and control groups at 4 weeks - dosage subgroup.**

**
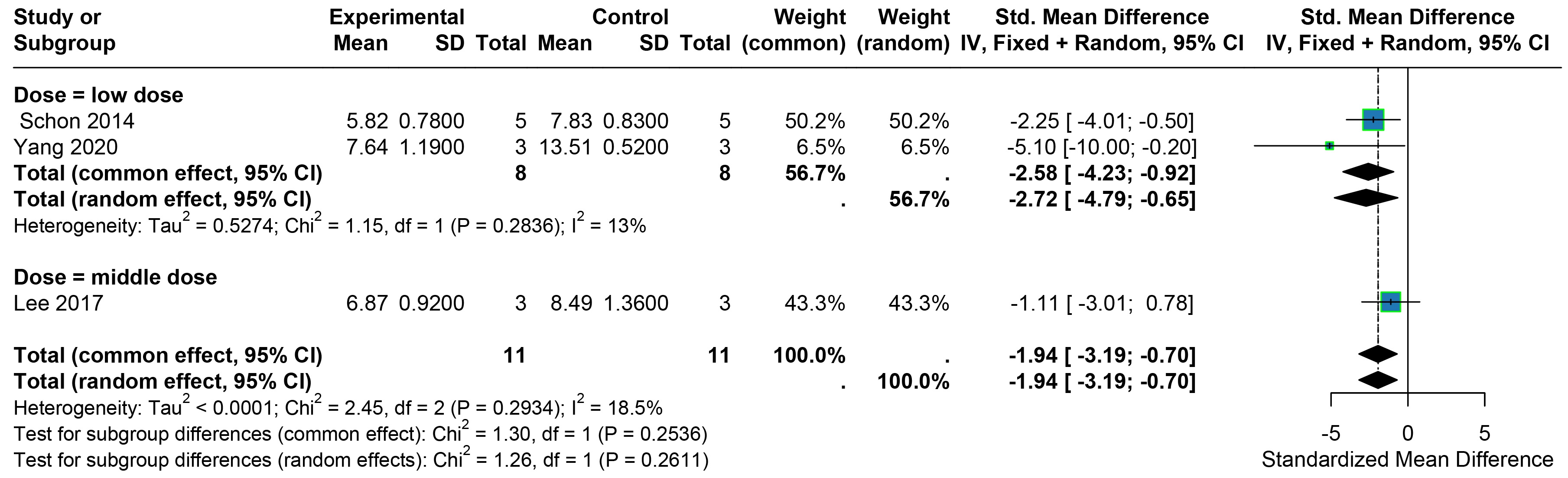
**

**Supplementary Figure 13: Histological scores comparison between Scaffold + Stem Cells and Scaffold groups at 2 weeks - dosage subgroup.**

**
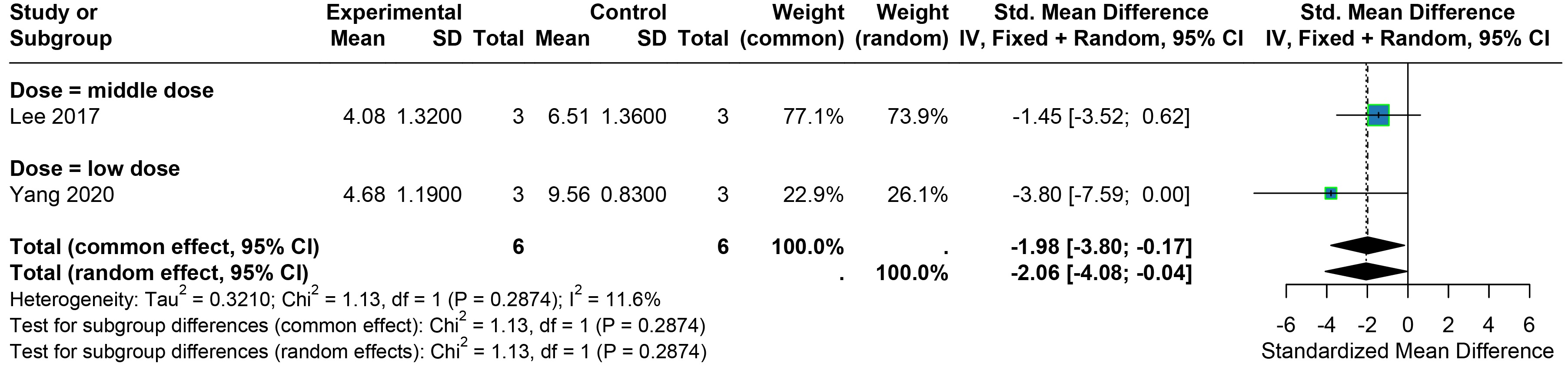
**

**Supplementary Figure 14: Histological scores comparison between Scaffold + Stem Cells and Scaffold groups at 4 weeks - dosage subgroup.**

**
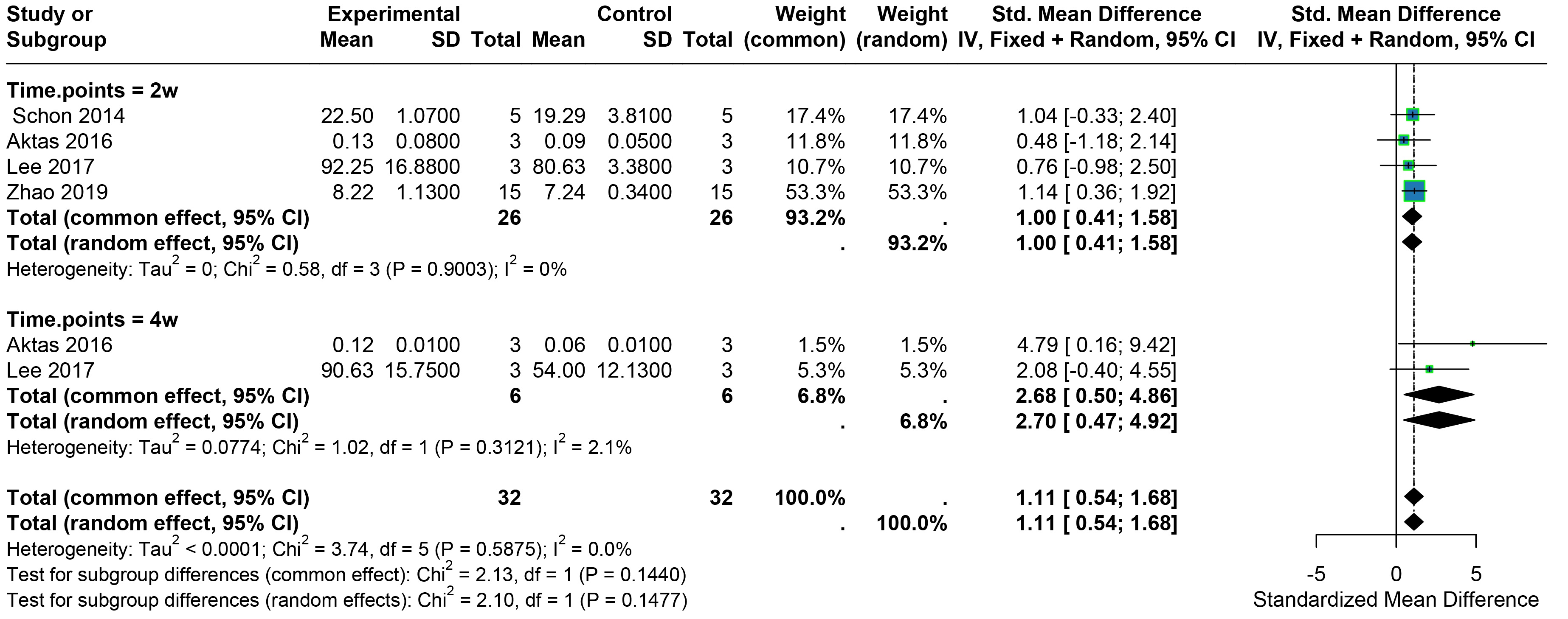
**

**Supplementary Figure 15: Type I collagen expression comparison between Scaffold + Stem Cells and Scaffold groups.**

**
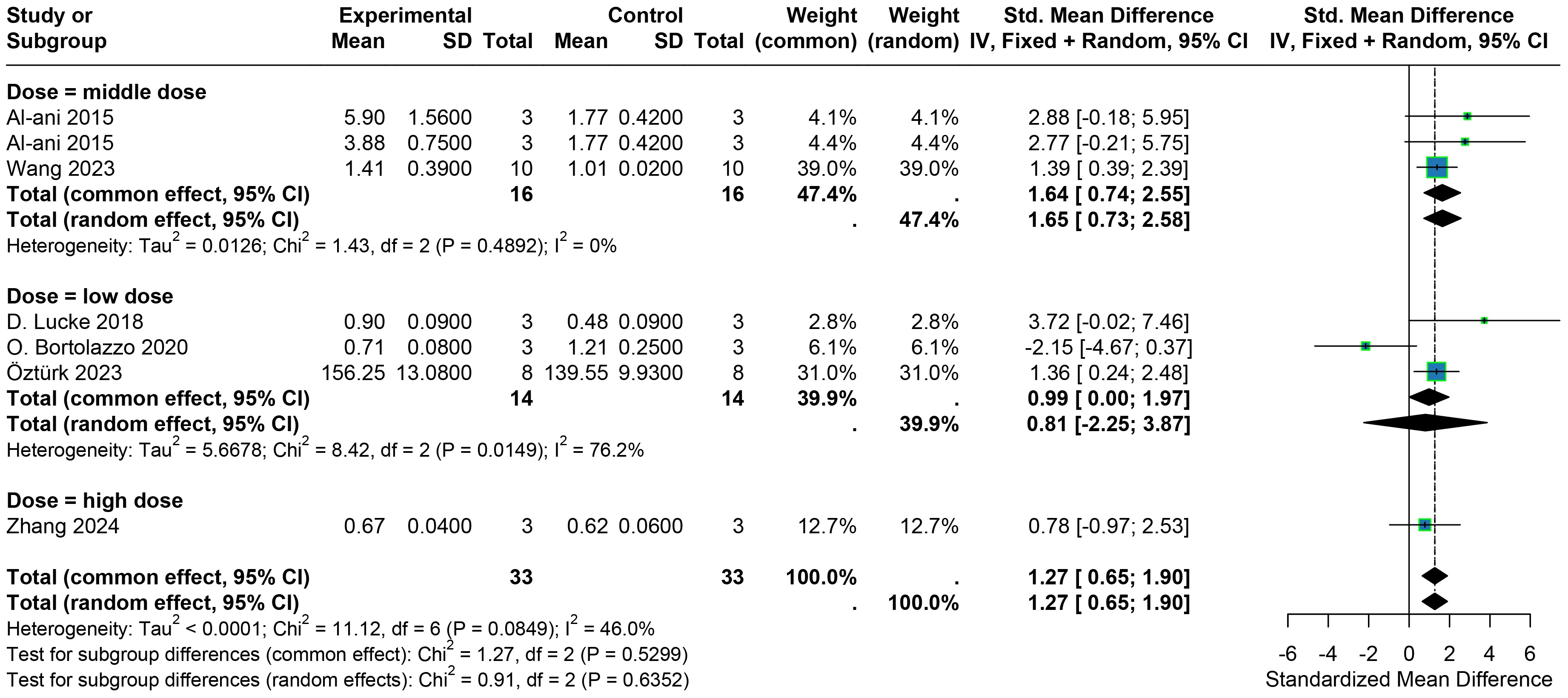
**

**Supplementary Figure 16: Type I collagen expression comparison between stem cells and control groups at 2 weeks - dosage subgroup.**

**
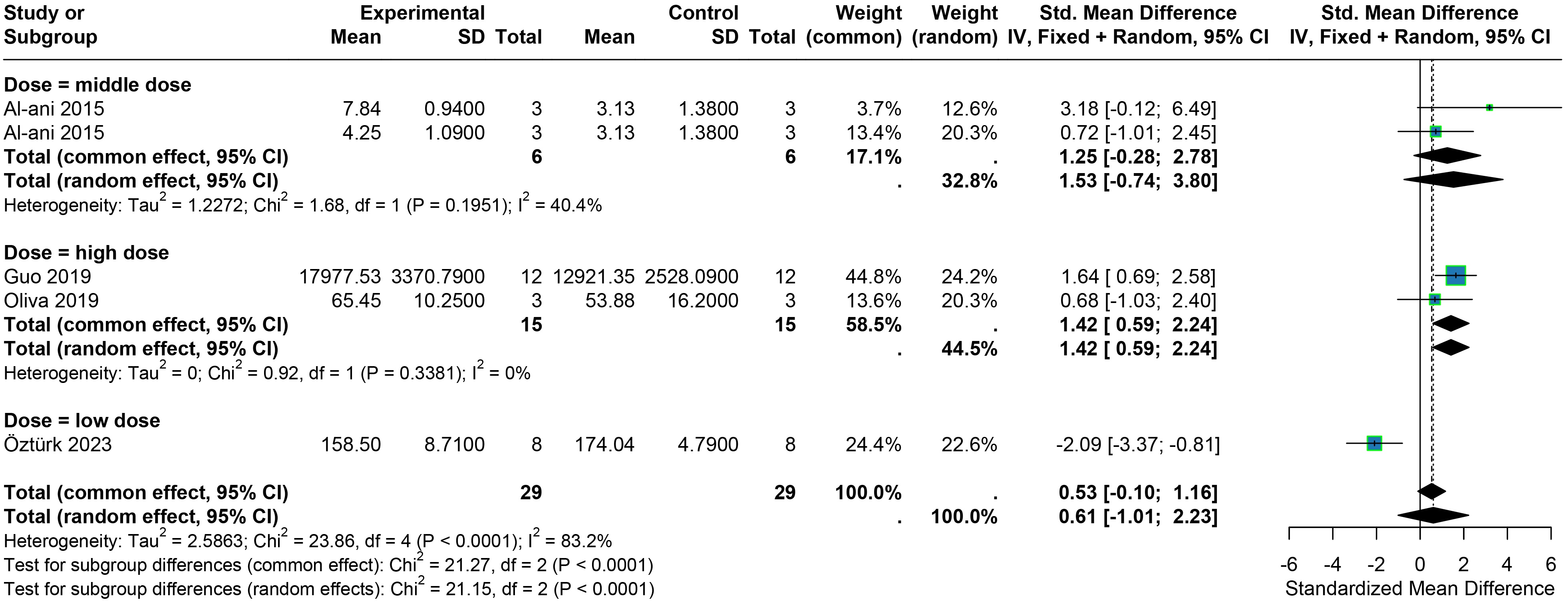
**

**Supplementary Figure 17: Type I collagen expression comparison between stem cells and control groups at 4 weeks - dosage subgroup.**

**
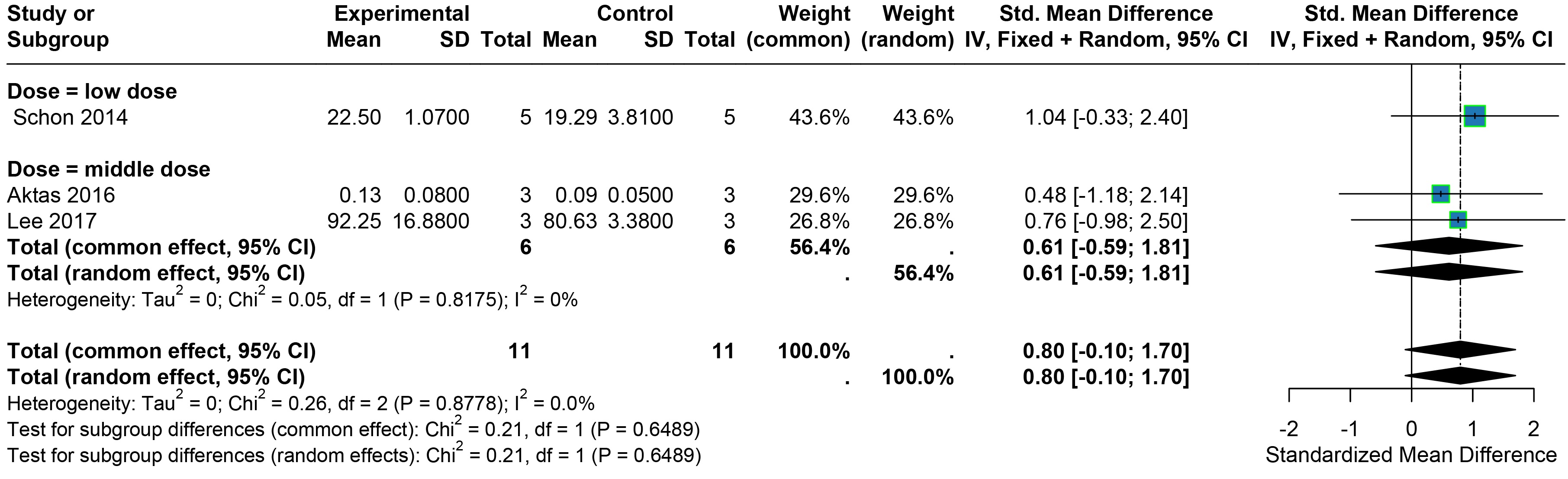
**

**Supplementary Figure 18: Type I collagen expression comparison between Scaffold + Stem Cells and Scaffold groups at 2 weeks - dosage subgroup.**

**
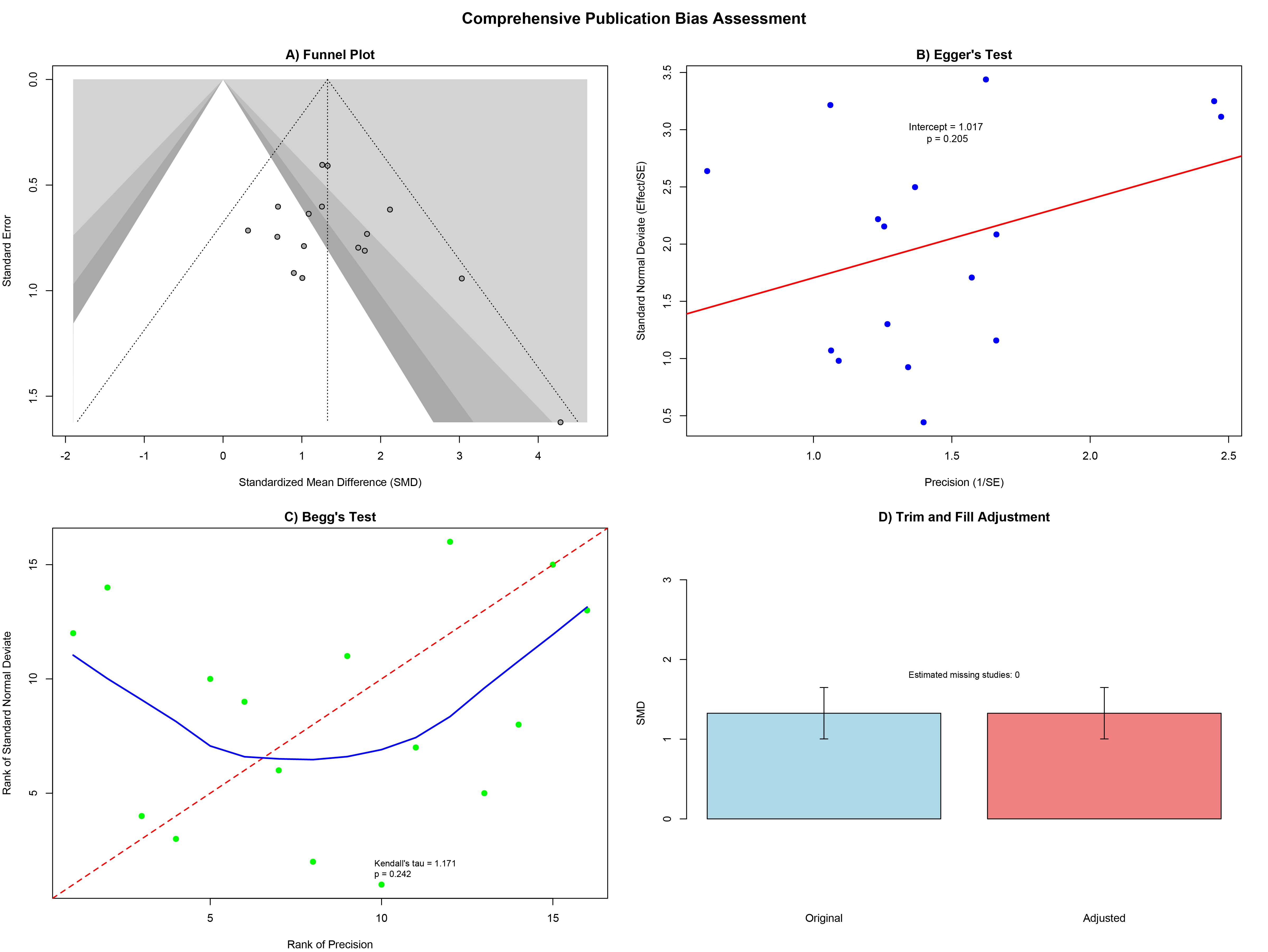
**

**Supplementary Figure 19: Publication bias assessment for maximum load comparison between Scaffold + Stem Cells and Scaffold groups.**
